# Prime editor‐mediated functional reshaping of *ACE2* prevents the entry of multiple human coronaviruses, including SARS‐CoV‐2 variants

**DOI:** 10.1002/mco2.356

**Published:** 2023-09-10

**Authors:** Wenwen Zhao, Jifang Li, Xiao Wang, Wei Xu, Bao‐Qing Gao, Jiangchao Xiang, Yaofeng Hou, Wei Liu, Jing Wu, Qilian Qi, Jia Wei, Xiaoyu Yang, Lu Lu, Li Yang, Jia Chen, Bei Yang

**Affiliations:** ^1^ Shanghai Frontiers Science Center for Biomacromolecules and Precision Medicine Shanghai Institute for Advanced Immunochemical Studies and School of Life Science and Technology ShanghaiTech University Shanghai China; ^2^ Gene Editing Center School of Life Science and Technology ShanghaiTech University Shanghai China; ^3^ Shanghai Clinical Research and Trial Center Shanghai China; ^4^ Center for Excellence in Molecular Cell Science Shanghai Institute of Biochemistry and Cell Biology Chinese Academy of Sciences Shanghai China; ^5^ Key Laboratory of Medical Molecular Virology (MOE/NHC/CAMS) School of Basic Medical Sciences Fudan University Shanghai China; ^6^ Shanghai Institute of Infectious Disease and Biosecurity, Fudan University Shanghai China; ^7^ Biosafety Level 3 Laboratory Shanghai Medical College Shanghai Frontiers Science Center of Pathogenic Microbes and Infection Fudan University Shanghai China; ^8^ Shanghai Institute of Nutrition and Health University of Chinese Academy of Sciences Chinese Academy of Sciences Shanghai China; ^9^ School of Physical Science and Technology ShanghaiTech University Shanghai China; ^10^ Center for Molecular Medicine Children's Hospital Fudan University Shanghai China; ^11^ Shanghai Key Laboratory of Medical Epigenetics International Laboratory of Medical Epigenetics and Metabolism Ministry of Science and Technology Institutes of Biomedical Sciences Fudan University Shanghai China

**Keywords:** broad spectrum, HCoV‐NL63, host factor reshaping, prime editing, SARS‐CoV, SARS‐CoV‐2 VOCs

## Abstract

The spike protein of SARS‐CoV‐2 hijacks the host angiotensin converting enzyme 2 (ACE2) to meditate its entry and is the primary target for vaccine development. Nevertheless, SARS‐CoV‐2 keeps evolving and the latest Omicron subvariants BQ.1 and XBB have gained exceptional immune evasion potential through mutations in their spike proteins, leading to sharply reduced efficacy of current spike‐focused vaccines and therapeutics. Compared with the fast‐evolving spike protein, targeting host ACE2 offers an alternative antiviral strategy that is more resistant to viral evolution and can even provide broad prevention against SARS‐CoV and HCoV‐NL63. Here, we use prime editor (PE) to precisely edit *ACE2* at structurally selected sites. We demonstrated that residue changes at Q24/D30/K31 and/or K353 of ACE2 could completely ablate the binding of tested viruses while maintaining its physiological role in host angiotensin II conversion. PE‐mediated *ACE2* editing at these sites suppressed the entry of pseudotyped SARS‐CoV‐2 major variants of concern and even SARS‐CoV or HCoV‐NL63. Moreover, it significantly inhibited the replication of the Delta variant live virus. Our work investigated the unexplored application potential of prime editing in high‐risk infectious disease control and demonstrated that such gene editing‐based host factor reshaping strategy can provide broad‐spectrum antiviral activity and a high barrier to viral escape or resistance.

## Graphical abstract

*ACE2*‐editing provided protection against multiple human coronaviruses (HCoVs), including SARS‐CoV, HCoV‐NL63, SARS‐CoV‐2, and its variants of concern. Model for *ACE2*‐editing‐provided protection against multiple HCoVs. Left: Wild‐type angiotensin converting enzyme 2 (ACE2) catalyzes the conversion of Ang II into Ang 1–7 and serves as the entry receptor for multiple HCoVs. Middle: *ACE2*‐editing site screening is guided by structural analysis, and editing outcomes are evaluated at multiple levels. Right: Precise editing of *ACE2* blocks the cell entry of multiple HCoVs and meanwhile maintains the physiological function of ACE2 in renin–angiotensin system.

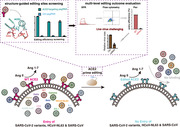

## 1 INTRODUCTION

Since its outbreak in 2019, COVID‐19 has ravaged the world for 3 years and caused nearly 7 million deaths worldwide (https://covid19.who.int). The high infectivity and substantial threat brought by SARS‐CoV‐2, the causative pathogen of COVID‐19, have attracted extensive efforts to develop preventives and therapeutics against its infection, among which vaccination has been one of the most effective strategies so far. Nevertheless, SARS‐CoV‐2 keeps evolving under selection pressures from herd immunity and new SARS‐CoV‐2 variants with enhanced infectivity, transmissibility, and immune evasion capabilities continue to emerge, rendering the vaccination less and less effective in preventing infections.^1^ Indeed, different SARS‐CoV‐2 variants of concern (VOCs) including Alpha, Beta, Gamma, Delta, and Omicron have driven recurrent waves of infection worldwide, and two latest Omicron subvariants BQ.1 and XBB manifested exceptional transmissibility and immune escape potential, even compared with earlier Omicron subvariants.^2^, ^3^ Meanwhile, millions of people with compromised immune systems may not obtain enough protection from vaccination. Indeed, recent clinical studies have reported that organ transplant recipients showed impaired response to vaccines.^4^, ^5^ Thus, the development of alternative prophylactic or therapeutic strategies against SARS‐CoV‐2 infection is important.

Successful viral infection entails a range of interactions between viral proteins and host factors. Hence, antiviral strategies can be directed against either viral proteins or host factors. Compared with targeting ever‐evolving viral proteins, targeting host factors offers an antiviral strategy that is more resistant to viral evolution and can even provide broad‐spectrum antiviral effects against different viruses as long as the targeted host factors play key roles in these viruses.^6^ However, most host factors possess important physiological functions.^7^ Hence, precise manipulation of host factors to prevent them from being hijacked by viruses while maintaining their physiological functions is key to the success of this host‐targeting strategy, which nevertheless remains highly challenging for traditional small molecule drug development efforts or gene editing tools that are more adept at gene disruption (e.g., Cas nucleases or base editors).^8^, ^9^, ^10^, ^11^ Recently, by combining reverse transcriptase (RTase) with the CRISPR‒Cas9 system, prime editor (PE) and its improved versions were reported to induce all types of base substitutions and small insertions or deletions (indels) in the genome with high efficiency, product purity, and specificity, thereby providing potential tools to precisely reshape host factors for antiviral purposes.^12^, ^13^, ^14^, ^15^, ^16^, ^17^, ^18^

The spike protein of SARS‐CoV‐2 hijacks the host angiotensin converting enzyme 2 (ACE2) to meditate its entry.^19^ As the major target of selection pressure from infection‐ or vaccine‐induced immunity, the spike region undergoes more mutations than other regions of the SARS‐CoV‐2 viral genome during the sustained transmission and evolution of SARS‐CoV‐2.^20^ In addition to developing preventives or treatments that target the fast‐evolving spike protein, precise functional reshaping of the host peptidase ACE2 through PE‐mediated editing at structurally defined sites may also confer efficient viral entry inhibition and leave the physiological function of ACE2 in the renin–angiotensin system (RAS) undisturbed at the meantime. Moreover, such a host‐targeting strategy would be more resistant to viral evolution and may provide broad prevention against not only SARS‐CoV‐2 and its different VOCs, but also SARS‐CoV and HCoV‐NL63, two other human coronaviruses (HCoVs), given that they all use ACE2 as their entry factor and exploit similar regions on the surface of ACE2 that are distant from its peptidase activity center.^21^, ^22^, ^23^, ^24^, ^25^, ^26^, ^27^

Here, we took host factor ACE2 as an example to demonstrate the potential application of PE in viral infection control and the advantages of such host factor reshaping strategies. With the aid of structure‐guided editing site selection, we used PE to introduce desired amino acid changes at Q24, D30, K31, and/or deletion at K353 in the endogenously expressed ACE2. Such editing of *ACE2* preserves its peptidase activity in RAS but completely ablates its interaction with the spike proteins from not only multiple SARS‐CoV‐2 VOCs, but also SARS‐CoV and HCoV‐NL63. Furthermore, by examining the entry of pseudovirus (PsV) and the replication of live virus in cells, our results showed that PE‐mediated editing of *ACE2* provides broad and effective prevention against all ACE2‐binding HCoVs, including multiple SARS‐CoV‐2 strains, SARS‐CoV, and HCoV‐NL63. Our work thus provided the first example to demonstrate the applicability and broad‐spectrum advantages of prime editing in host factor reshaping for antiviral purposes.

## 2 RESULTS

### 2.1 Structure‐guided site selection for *ACE2* editing

**FIGURE 1:**
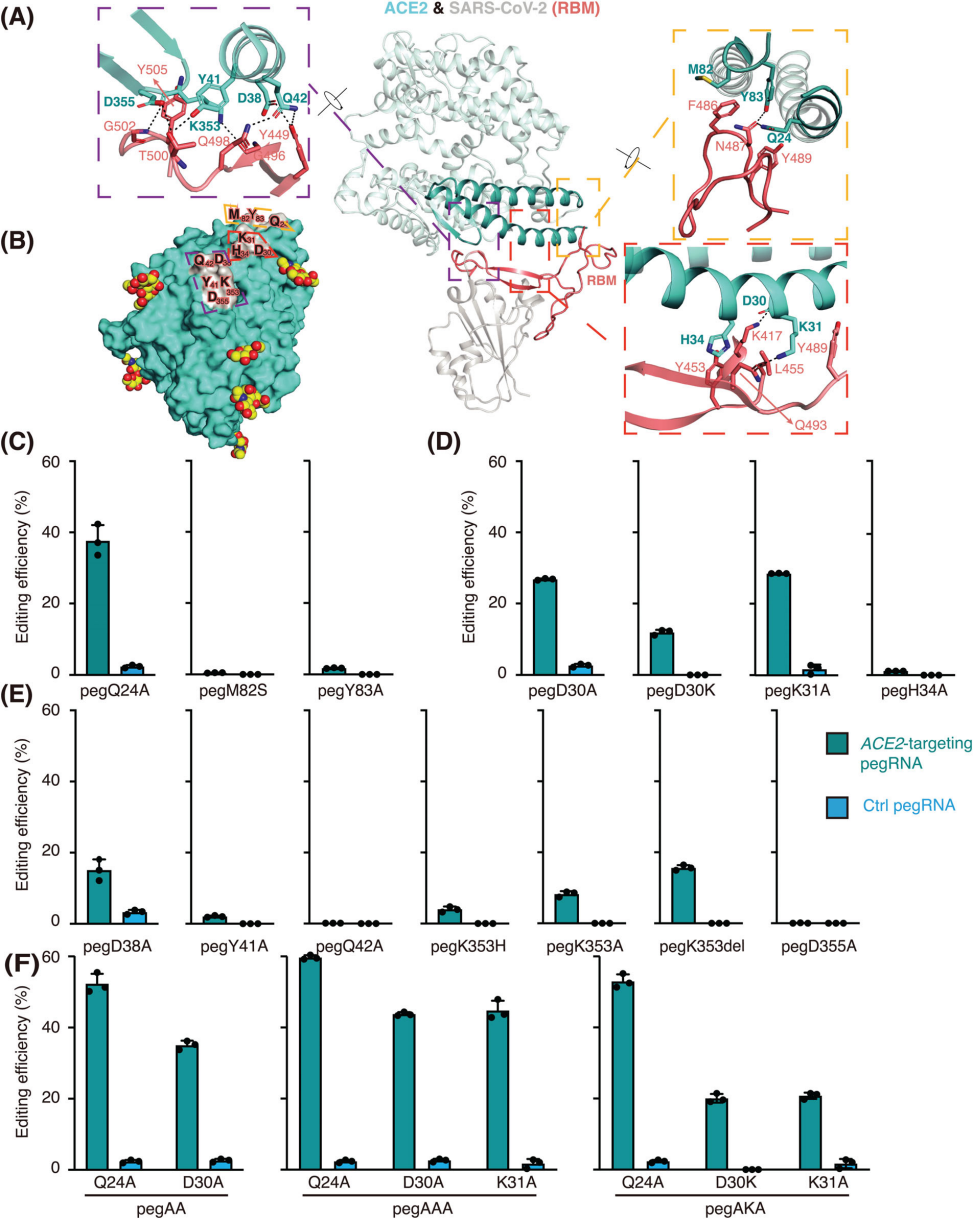
Structure‐guided editing site selection and evaluation. (A) A representative structure (PDBid: 6m17) of angiotensin converting enzyme 2 (ACE2) (light cyan) in complex with SARS‐CoV‐2 receptor‐binding domain (RBD) (gray) is shown as ribbons. The interface mainly involves the receptor‐binding motif (salmon, labeled RBM) in the SARS‐CoV‐2 RBD, as well as the α1–α2 helices and β3–β4 loop in ACE2 (cyan), wherein consensus interface residues on ACE2 are further grouped into three clusters and detailed in close‐up views (Clusters 1−3 depicted with orange, red, and purple dashed boxes, respectively). Key contacting residues are shown as stick models, and polar and electrostatic interactions are indicated with black dashed lines. (B) Foot‐printing of RBD (gray) on the surface presentation of ACE2 (cyan). ACE2 residues involved in binding are labeled and boxed as in (A) to illustrate the three clusters. N‐glycans on ACE2 are depicted as red and yellow spheres. (C–E) Efficiency of prime editor (PE)‐mediated editing of individual ACE2 residues from Clusters 1 (C), 2 (D), and 3 (E). (F) Efficiency of PE‐mediated simultaneous editing of ACE2 residues from Clusters 1 and 2 with one pegRNA.

When selecting potential sites for *ACE2* editing, we considered the structure of full‐length ACE2 in complex with SARS‐CoV‐2 receptor‐binding domain (RBD) (PDBid: 6m17), as well as that of the intact SARS‐CoV‐2 spike protein in complex with ACE2 peptidase domain (PDBid: 7kj4), to identify residues that are most determinant for the interaction between ACE2 and the spike protein.^28^, ^29^ Only ACE2 residues that consistently bind the SARS‐CoV‐2 RBD in both structures were considered consensus interface residues and were chosen for editing (Figure S1). These residues fall into three clusters on the interface and include residues Q24, M82, and Y83 from Cluster 1, D30, K31, and H34 from Cluster 2, and D38, Y41, Q42, K353, and D355 from Cluster 3 (Figure 1A,B).

Using PE3, we then edited these residues into amino acids that may disrupt the binding of the SARS‐CoV‐2 RBD, either individually (Figure 1C–E) or simultaneously if possible (Figure 1F). We found that the editing efficiencies varied from 0% to 60% (Figure 1C–F). Among all tested prime editing guide RNAs (pegRNAs), pegQ24A, pegAA (for Q24A/D30A), pegAAA (for Q24A/D30A/K31A), and pegAKA (for Q24A/D30K/K31A) induced efficient genome editing (maximal efficiency >30%) for Cluster 1 and/or 2 residue changes (Figure 1C,F). In Cluster 3, pegK353del induced relatively efficient editing (∼20% efficiency) of the K353 deletion (Figure 1E). Thus, these sites and their combinations were chosen for subsequent evaluation.

### 2.2 Preliminary evaluation of *ACE2* editing outcome

**FIGURE 2:**
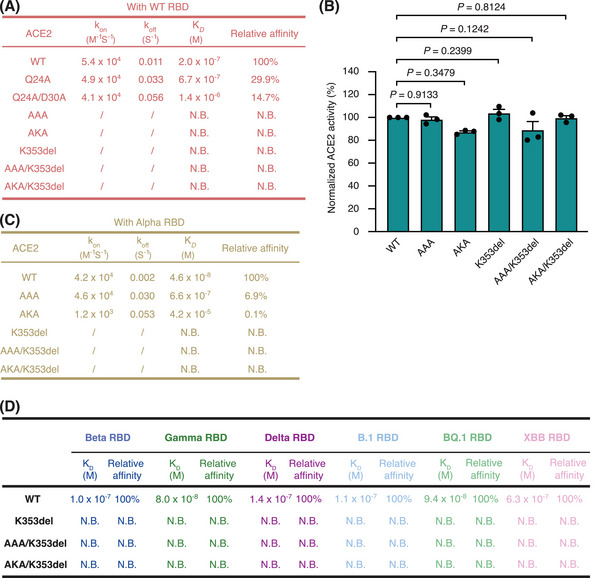
Specific mutations rendered angiotensin converting enzyme 2 (ACE2) resistant to the binding of the receptor‐binding domains (RBDs) from different SARS‐CoV‐2 strains. (A) Interactions between the ectodomains of the indicated ACE2 isoforms and immobilized SARS‐CoV‐2 RBD (wild‐type [WT] strain) were determined via surface plasmon resonance (SPR), wherein AAA (Q24A/D30A/K31A), AKA (Q24A/D30K/K31A), K353del, AAA/K353del, and AKA/K353del completely eliminated the interaction between the two. N.B. denotes no binding. (B) The Ang II hydrolysis activity of ACE2ecd was not significantly affected by the indicated mutations. Data are shown as the mean ± standard deviation (SD) (*n* = 3). *p*‐Values were determined by a *t* test. (C and D) Interactions between the ectodomains of the indicated ACE2 isoforms and the immobilized RBD proteins of SARS‐CoV‐2 variants of concern (VOCs) Alpha (C), Beta (D), Gamma (D), Delta (D), and Omicron (including B.1, BQ.1, and XBB subvariants) (D) were determined with SPR. Edited ACE2 isoforms K353del, AAA/K353del, and AKA/K353del are resistant to the binding of RBDs from all VOCs tested. N.B. denotes no binding.

We first characterized the binding between the wild‐type SARS‐CoV‐2 RBD (WT RBD) and the extracellular protease domain of wild‐type ACE2 (WT ACE2ecd) or its PE3‐edited isoforms via surface plasmon resonance (SPR). WT ACE2ecd bound to immobilized WT RBD with an equilibrium disassociation constant (*K*_D_) of 200 nM, while ACE2ecd isoforms Q24A and Q24A/D30A bound to WT RBD with *K*_D_ values of 670 nM and 1.4 μM, respectively, each representing a 70.1% and 85.3% reduction in affinity (Figures 2A and S2A). Moreover, ACE2ecd isoforms AAA (Q24A/D30A/K31A), AKA (Q24A/D30K/K31A), K353del, AAA/K353del, and AKA/K353del did not bind WT RBD at all (Figures 2A and S2A), suggesting that corresponding editing at these sites may prevent ACE2 from serving as the receptor of WT SARS‐CoV‐2.

In addition to acting as the entry point for certain CoVs, the primary physiological function of ACE2 is to catalyze the conversion of Ang II, a vasoconstrictor, into angiotensin (1–7), a vasodilator, thereby counteracting the activity of ACE to maintain blood pressure stability and fluid or electrolyte balance.^30^ Hence, when editing *ACE2* to prevent it from being hijacked by CoVs as the entry point, it was essential not to disturb its Ang II hydrolysis function. We thus measured the catalytic activity of WT ACE2ecd and the five edited isoforms that no longer bound the WT RBD (Figure 2B). The results indicate that the enzyme activity of ACE2ecd was not significantly affected by these mutations (Figure 2B), which was consistent with the observation that these edited ACE2 isoforms did not undergo any major conformational changes in their protease domain as compared to WT ACE2 (Figure S3).

All SARS‐CoV‐2 VOCs contained mutations in the RBD of the spike protein (Figure S2B), raising the possibility of an altered ACE2 interaction mode. To test whether the editing of *ACE2* would still prevent it from being bound by these SARS‐CoV‐2 VOCs, we also investigated the binding kinetics and affinity of WT ACE2ecd or its edited isoforms to corresponding RBDs (Figure 2C,D). WT ACE2ecd bound to the immobilized alpha RBD with a *K*_D_ of 46 nM, consistent with previous reports that WT ACE2ecd has a higher affinity for the alpha RBD than for the WT RBD (Figure 2C).^31^ Of the five ACE2ecd isoforms that did not bind the WT RBD, isoforms AAA and AKA bound the alpha RBD with *K*_D_ values of 660 nM and 42 μM, respectively, while isoforms K353del, AAA/K353del, and AKA/K353del did not show any binding to the alpha RBD (Figures 2C and S4). These results indicated that isoforms K353del, AAA/K353del, and AKA/K353del may possess broad resistance to the binding of RBDs from different SARS‐CoV‐2 VOCs. Indeed, although WT ACE2ecd bound the immobilized RBDs from Beta, Gamma, Delta, Omicron B.1, Omicron BQ.1, and Omicron XBB with *K*_D_ values of 100, 80, 140, 110, 94, and 630 nM, respectively, essentially no binding was observed between the above three ACE2ecd isoforms and these RBDs (Figures 2D and S4).

**FIGURE 3:**
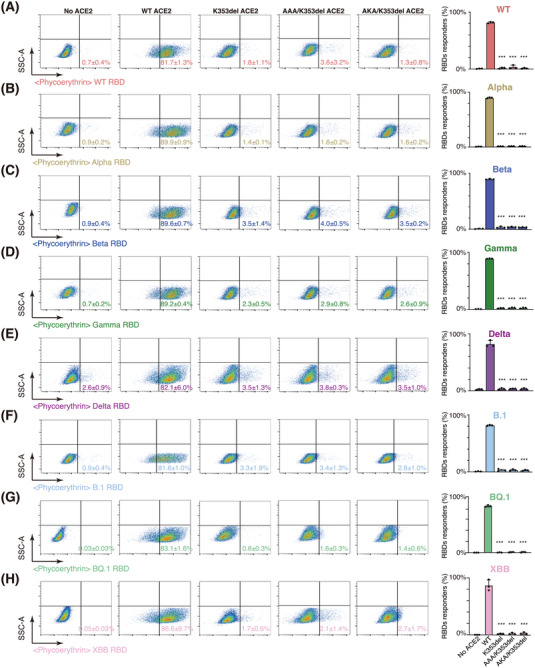
Cell surface interaction between edited angiotensin converting enzyme 2 (ACE2) isoforms and the receptor‐binding domains (RBDs) from different SARS‐CoV‐2 strains. (A–H) Representative flow cytometry plots (left five panels) showing the cell surface interaction of full‐length wild‐type (WT) ACE2 or its edited isoforms with the RBDs from globally prevalent SARS‐CoV‐2 strains, including WT (A), Alpha (B), Beta (C), Gamma (D), Delta (E), Omicron B.1 (F), Omicron BQ.1 (G), or Omicron XBB (H). ACE2‐KO 293FT cells (no ACE2 expression) were used as a negative control, while ACE2‐KO 293FT cells exogenously overexpressing WT ACE2 (WT) served as positive controls. The proportion of cells exhibiting a detectable response to the RBD from each indicated SARS‐CoV‐2 strain is shown in the far‐right panel. Data are shown as the mean ± standard deviation (SD) (*n* = 3). *p*‐Values were determined by comparing with the WT group through a *t* test. ^*^ *p* < 0.05, ^**^ *p* < 0.01, ^***^ *p* < 0.001.

Next, we evaluated the binding capacity of different SARS‐CoV‐2 RBDs to full‐length ACE2 or its edited isoforms through a flow cytometry‐based assay. We first established a 293FT cell line wherein endogenous *ACE2* was knocked out (hereafter referred to as ACE2‐KO 293FT cells) (Figure S5A–C). Then, we exogenously overexpressed WT ACE2 or its edited isoforms K353del, AAA/K353del, and AKA/K353del in ACE2‐KO 293FT cells and confirmed that the ACE2 protein expression levels and Ang II converting activities were similar among these cells (Figure S5D,E). Consistent with the biochemical evaluation (Figure 2A,C,D), while WT‐ACE2‐expressing cells were efficiently responsive to WT, Alpha, Beta, Gamma, Delta, and Omicron (including B.1, BQ.1, and XBB subvirants) RBDs, cells expressing full‐length ACE2 with K353del, AAA/K353del, or AKA/K353del mutations were not responsive to the RBDs from any of the tested SARS‐CoV‐2 strains (Figure 3).

### 2.3 *ACE2* editing blocks the entry of pseudotyped SARS‐CoV‐2 VOCs

**FIGURE 4:**
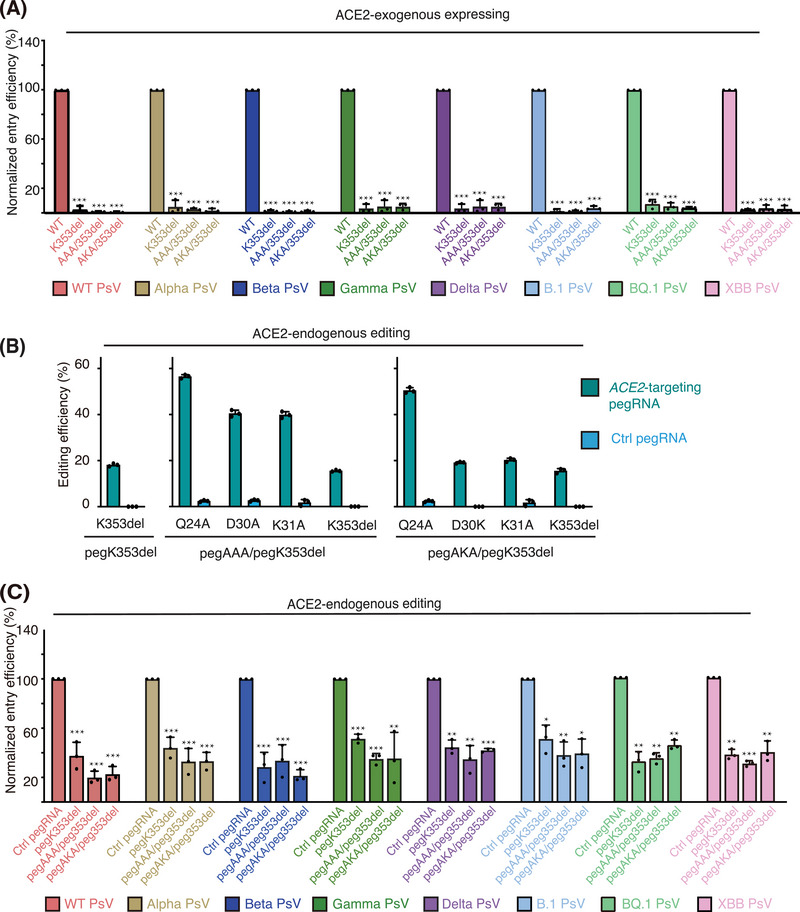
*ACE2* editing prevented the entry of different pseudotyped SARS‐CoV‐2 strains. (A) ACE2‐KO 293FT cells exogenously overexpressing wild‐type (WT) angiotensin converting enzyme 2 (ACE2) or its edited isoforms were challenged with different pseudoviruses (PsVs), and the entry efficiency of the corresponding PsV into cells exogenously overexpressing WT ACE2 was set as 100%. (B) The efficiencies of prime editor (PE)‐mediated *ACE2* editing at the indicated target sites in EF1αP‐KI 293FT cells, which express endogenous ACE2 at a high level. (C) EF1αP‐KI 293FT cells were edited by PE at the indicated sites and then challenged with different PsVs. The entry efficiency of the corresponding PsV into mock‐edited EF1αP‐KI 293FT cells was set as 100%. (A and C) Eight types of PsVs each correspond to the SARS‐CoV‐2 WT, Alpha, Beta, Gamma, Delta, Omicron B.1, Omicron BQ.1, and Omicron XBB strains. PsV entry efficiency was characterized as luciferase activity accompanying entry. Data are shown as the mean ± standard deviation (SD) (*n* = 3). *p*‐Values were determined by comparing with the WT group (A) or Ctrl pegRNA group (C) through a *t* test. ^*^ *p* < 0.05, ^**^ *p* < 0.01, ^***^ *p* < 0.001.

Next, we investigated the resistance of these K353del‐, AAA/K353del‐, or AKA/K353del‐expressing cells to SARS‐CoV‐2 infection through PsV experiments. Pseudotyped SARS‐CoV‐2 VOCs were used for infection, and the results indicated that cells exogenously expressing the edited ACE2 isoforms K353del, AAA/K353del, or AKA/K353del were entirely resistant to infection by all tested SARS‐CoV‐2 strains (Figure 4A).

Then, we sought to examine whether PE3‐mediated editing of endogenous *ACE2* can provide cell resistance to different SARS‐CoV‐2 variants. As the expression of endogenous ACE2 in 293FT cells is low, we first knocked in an elongation factor‐1 alpha (EF1α) promoter upstream of the transcription start site of *ACE2* to upregulate its expression (Figure S6A–C). We then edited the EF1α‐promoter‐knock‐in (EF1αP‐KI) 293FT cells by using pegK353del only, pegAAA/pegK353del, or pegAKA/pegK353del and found that these pegRNAs all induced efficient editing at the target sites (Figure 4B), and no observable mutation was triggered at the predicted off‐target sites (Figure S6D–I).^32^ As expected, the expression of ACE2 proteins and cellular Ang II converting activities were not significantly altered by endogenous *ACE2* editing (Figure S6J,K). Next, these *ACE2*‐edited EF1αP‐KI cells were challenged with pseudotyped SARS‐CoV‐2 VOCs and exhibited entry inhibition of all SARS‐CoV‐2 strains, as compared to mock‐edited cells (Ctrl pegRNA) (Figure 4C). Altogether, these results indicated that PE‐mediated genome editing of *ACE2* at selected sites could provide broad and effective prevention against the entry of different SARS‐CoV‐2 strains.

### 2.4 Broad protection against other HCoVs by *ACE2* editing

The spike proteins of SARS‐CoV‐2, SARS‐CoV, and HCoV‐NL63 target overlapping regions on the surface of ACE2 for binding (Figure S7A–C). Among all of the spike‐contacting residues on the surface of ACE2, interface residues H34, Y41, and K353 are shared between these three HCoVs (Figure S7A–C, labeled with red frames). Although the editing of H34 and Y41 was inefficient (Figure 1D,E), the deletion of K353 was successfully achieved (Figures 1E and 4B) and manifested a broad blocking effect on the invasion of various SARS‐CoV‐2 strains (Figure 4). These results prompted us to test whether *ACE2* editing at K353 would exhibit even broader anti‐HCoV effects on SARS‐CoV and HCoV‐NL63.

**FIGURE 5:**
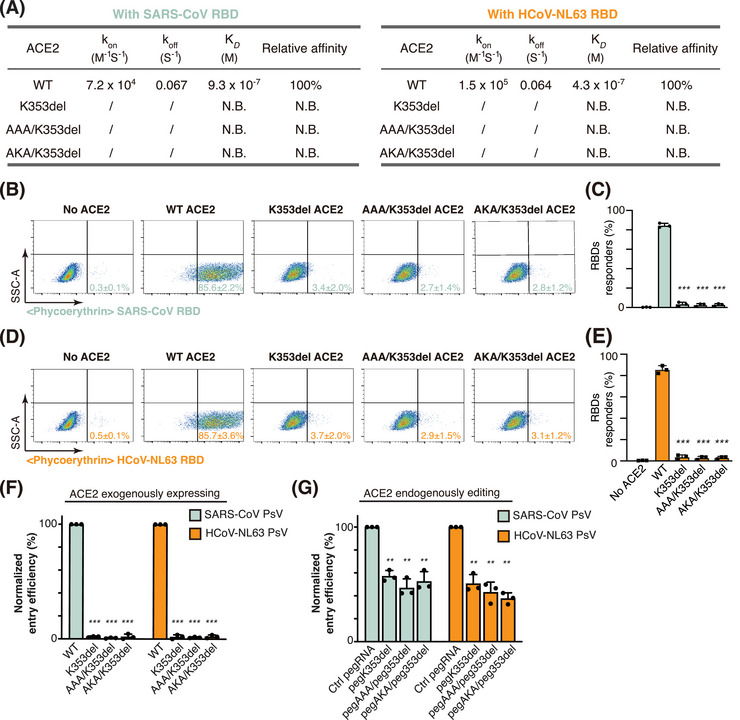
Broad‐spectrum virus entry prevention by *ACE2* editing. (A) Isoforms K353del, AAA/K353del, and AKA/K353del rendered angiotensin converting enzyme 2 (ACE2) resistant to the binding of the receptor‐binding domains (RBDs) from SARS‐CoV and HCoV‐NL63, suggesting the broad‐spectrum antiviral effects of these mutations. N.B. denotes no binding. (B and D) Representative flow cytometry plots showing the cell surface interaction of full‐length wild‐type (WT) ACE2 or its edited isoforms with the RBDs from SARS‐CoV (B) or HCoV‐NL63 (D). ACE2‐KO 293FT cells (no ACE2) were used as a negative control, while ACE2‐KO 293FT cells overexpressing WT ACE2 (WT ACE2) served as positive controls. (C and E) Proportion of cells exhibiting a detectable response to RBDs from SARS‐CoV (C) or HCoV‐NL63 (E). Data are shown as the mean ± standard deviation (SD) (*n* = 3). *p*‐Values were determined by a *t* test. (F) ACE2‐KO 293FT cells exogenously overexpressing WT ACE2 or its edited isoforms were challenged with SARS‐CoV or HCoV‐NL63 pseudoviruse (PsV), and the entry efficiency of the corresponding PsVs into cells exogenously overexpressing WT ACE2 was set as 100%. (G) Endogenous *ACE2* was edited by prime editor (PE) at the indicated sites in EF1αP‐KI 293FT cells before SARS‐CoV or HCoV‐NL63 PsV challenge. The entry efficiency of corresponding PsVs into mock‐edited cells was set as 100%. (F and G) PsV entry efficiency was characterized as luciferase activity accompanying entry. Data are shown as the mean ± SD (*n* = 3). (C, E, F, and G) *p*‐Values were determined by comparing with the WT group (C, E, F) or Ctrl pegRNA group (G) through a *t* test. ^*^ *p* < 0.05, ^**^ *p* < 0.01, ^***^ *p* < 0.001.

SPR results showed that ACE2ecd with K353del, AAA/K353del, or AKA/K353del mutations no longer bound to the RBDs from SARS‐CoV and HCoV‐NL63 (Figures 5A and S7D,E). Consistently, the cells expressing full‐length ACE2 with these mutations were not responsive to the RBDs from SARS‐CoV and HCoV‐NL63 (Figure 5B–E). We next determined the entry of pseudotyped SARS‐CoV and HCoV‐NL63 into cells expressing WT ACE2 or its edited isoforms. The entry of SARS‐CoV and HCoV‐NL63 PsVs was almost completely blocked in cells expressing ACE2 K353del, AAA/K353del, or AKA/K353del isoforms compared with cells expressing WT ACE2 (Figure 5F). Moreover, the editing of endogenous *ACE2* significantly suppressed the entry of pseudotyped SARS‐CoV and HCoV‐NL63 (Figure 5G). Together, these data suggest that editing of *ACE2* at corresponding sites would prevent ACE2 from serving as the host receptor for SARS‐CoV and HCoV‐NL63.

### 2.5 *ACE2*‐editing‐provided protection against live virus infection

**FIGURE 6:**
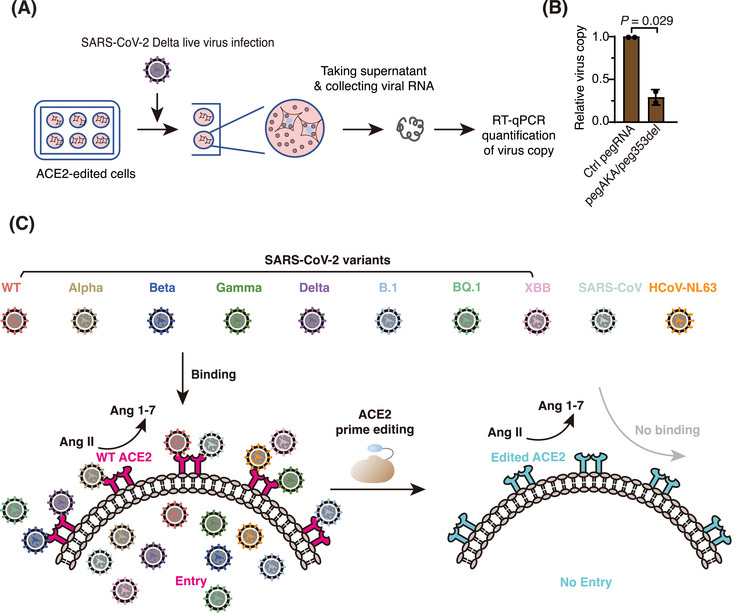
*ACE2*‐editing‐provided protection against multiple human coronaviruses (HCoVs). (A) Schematic diagram illustrating the SARS‐CoV‐2 variant of concern (VOC) Delta live virus challenge of *ACE2‐*edited EF1αP‐KI 293FT cells and characterization of live virus copies. (B) Live virus replication was characterized by RT‐PCR quantification of viral genomic RNA copies in the cell culture supernatant. The viral genomic RNA copies in mock‐edited wells were referred to as 100%. Data are shown as the mean ± standard deviation (SD) (*n* = 2). *p*‐Values were determined by comparing with the Ctrl pegRNA group through a *t* test. (C) A model for *ACE2*‐editing‐provided protection against multiple HCoVs. Left: WT angiotensin converting enzyme 2 (ACE2) catalyzes the conversion of Ang II into Ang 1−7 and serves as the entry receptor for multiple HCoVs. Right: Precise editing of *ACE2* blocks the cell entry of multiple HCoVs and maintains the physiological function of ACE2 in renin–angiotensin system (RAS).

Encouraged by the results from PsV experiments, we further examined whether PE editing of *ACE2* can provide protection against live virus infection. We first edited endogenous *ACE2* with either control pegRNA (Ctrl pegRNA) or pegAKA/pegK353del and then challenged the edited cells with Delta live virus (Figure 6A). After 24 h, we quantified and compared the viral genomic RNA copies in the cell culture (Figure 6A). Compared to mock editing, editing of *ACE2* with pegAKA/pegK353del resulted in an ∼70% reduction in viral genomic RNA copies (Figure 6B). This result showed that PE‐mediated *ACE2* editing could also provide efficient protection against SARS‐CoV‐2 live virus infection.

## 3 DISCUSSION

While genome editing is widely accepted as a promising therapy for hereditary disorders, its potential in treating high‐risk infectious diseases (e.g., AIDS) by disrupting key host factors (e.g., C–C chemokine receptor type 5, CCR5) has only started to be explored recently.^33^ Unlike the CCR5 receptor, which has a Delta32 deletion heterozygous allele frequency of ∼10% in Europe and thus appears dispensable, most viral‐related host factors have important physiological functions.^34^ Hence, preserving such functions while ablating their roles in viral infections is imperative for the success of the host‐targeting strategy. Here, we used ACE2, the host receptor for SARS‐CoV‐2, as a model to demonstrate that such accurate functional reshaping of host factors can be accomplished by PE when combined with structure‐guided editing site selection. By employing PE, selected ACE2 residues were changed or deleted accurately and efficiently, which blocked the binding and entry of all tested ACE2‐binding HCoVs, including not only all tested SARS‐CoV‐2 variants but also SARS‐CoV and HCoV‐NL63 (Figure 6C). Moreover, the native function of ACE2 was not affected by editing (Figures 2B, S5E, and S6K). Our study thus demonstrates the applicability and broad‐spectrum advantages of using the recently developed PE method to reshape host factors for high‐risk infection control. The same strategy can be extended to the control of other human or animal viral infectious diseases and provide long‐lasting, broad‐spectrum antiviral effects, given that the physiological and viral‐related functions of the involved host factors can be separated and delineated structurally to guide editing site selection. For instance, the same strategy, when applied to livestock breeding through the embryonic editing of porcine aminopeptidase N (pAPN), could broadly prevent the entry of porcine deltacoronavirus, transmissible gastroenteritis virus (TGEV), and porcine respiratory coronavirus and exert profound economic impact on the domestic animal industry.^35^, ^36^, ^37^, ^38^

Interestingly, we noticed that a ∼20%–60% editing frequency of *ACE2* (Figure 4B) provided relatively high efficacy in blocking the entry of SARS‐CoV‐2 variants, SARS‐CoV and HCoV‐NL63 (Figures 4, 5, and 6B). Consistent with such findings, a recent study demonstrated that ACE2 downregulation reduces susceptibility to SARS‐CoV‐2 infection.^39^ Together, these observations imply that the efficient host entry of SARS‐CoV‐2 may require a high local level of ACE2 on the cell membrane, possibly due that complete disassociation of the S1 fragment and the freeing of the S2 membrane‐fusion apparatus require simultaneous or successive engagement of three ACE2 proteins with the trimeric spike.^40^

Recently, Li et al.^41^ utilized polymer‐formulated inhalable nanoparticles to deliver Cas9 mRNA and sgRNAs and achieved efficient CRISPR/Cas9 gene editing in lung tissues. As our prime editing system can also be delivered via mRNA and pegRNA, the same strategy can be applied for therapeutic PE delivery. Also, researchers have delivered polymer‐formulated RNA through nebulizers to achieve RNA editing in the respiratory tract and tested the efficacy of RNA editing in treating respiratory viral infections.^42^ Compared to RNA editing, the protection conferred by genome editing can last for a long period due to the permanent change in genomic DNA.^43^, ^44^ Meanwhile, no observable genome‐wide or transcriptome‐wide off‐target mutations were induced by PE, further corroborating the safety of its application in treating or preventing human or animal infectious diseases.^14^, ^15^, ^45^ However, to achieve highly efficient in vivo PE editing in respiratory tracts, high doses of mRNA and pegRNA need to be delivered, which would render the PE‐based ACE2 reshaping strategy proposed here too costly to afford for most people. Nevertheless, the potential application of this strategy in livestock breeding might be a viable outlet, which would help to curb the inter‐species jumping and unmonitored evolution of SARS‐CoV‐2. Meanwhile, using PE to perform saturated mutational scanning for protein function profiling has been reported, and the same method can be applied to *ACE2* gene as well to thoroughly characterize the function of its genetic variants.^46^ Moreover, new PE versions and optimized pegRNA design with artificial intelligence have also been developed to achieve higher editing efficiency or to ease its delivery for in vivo editing.^13^, ^18^, ^47^, ^48^, ^49^, ^50^ With the broad‐spectrum advantage of host‐targeting strategy and its high barrier to viral escape, we thus envision that PE‐mediated host factor reshaping could have broad applications in infectious disease control in the future, given the rapid development of in vivo delivery methods.

## 4 MATERIALS AND METHODS

### 4.1 Human cell lines and cell culture

All cell lines were cocultured at 37°C and 5% CO_2_. 293FT cells (R70007, Thermo Fisher Scientific) were maintained in DMEM (Dulbecco's Modified Eagle Medium, 10566, Gibco/Thermo Fisher Scientific), Calu‐3 cells (HTB‐55, ATCC) were maintained in MEM (Minimum Essential Medium, 11095, Gibco), and A549 cells (CRM‐CCL‐185, ATCC) were maintained in DMEM/F‐12 (11330, Gibco). All media were supplemented with 10% FBS (Fetal Bovine Serum, 16000‐044, Gibco). The cells have been tested to exclude mycoplasma contamination.

### 4.2 Plasmid construction

Primer sets (ACE2_PCR_F/ACE2_PCR_R) were used to amplify the full‐length WT human *ACE2* (hereafter referred as ACE2) gene from pUC57‐Human_ACE2 template (synthesized by GenScript). The amplified gene fragment was then cloned into the pcDNA3.1_pEF1α‐HA vector using ClonExpress II One Step Cloning kit (Vazyme, C112‐02) to generate the WT ACE2 expression plasmid pcDNA3.1_pEF1α‐ACE2‐HA. The expression plasmids of different ACE2 isoforms, including pcDNA3.1_pEF1α‐ACE2_K353del‐HA, pcDNA3.1_pEF1α‐ACE2_AAA/K353del‐HA, and pcDNA3.1_pEF1α‐ACE2_AKA/K353del‐HA, were all constructed through site‐directed mutagenesis and verified by DNA sequencing.

Two primer sets (U6_peg_F/U6_Q24_R) (U6_Q24A_F/ U6_Q24A_R1/R2) were used to amplify the U6‐peg‐Q24A fragment. Then, the fragment was cloned into the pGL3‐U6‐sgRNA‐PGK‐puromycin vector to generate the pegQ24A expression plasmid pGL3‐U6‐pegQ24A. Other pegRNA expression plasmids were constructed in similar ways. Oligonucleotides ACE2_nick_FOR/ACE2_nick_REV were annealed and ligated into BsaI linearized pGL3‐U6‐sgRNA‐PGK‐puromycin vector to generate the nicking sgRNA expression plasmids psgNickACE2. Other nicking sgRNA expression plasmids were constructed in the similar ways.

Three primer sets (KI_donor_F1/ KI_donor_R1), (KI_donor_F2/ KI_donor_R2), and (KI_donor_F3/ KI_donor_R3) were used to amplify the EF1α promoter fragment with ACE2 homologous sequence. Then, the fragment was cloned into XbaI and MluI linearized pUC57 vector to generate EF1α promoter‐containing donor plasmid (EF1αP donor).

The extracellular protease domain of ACE2 (residues 19−615, hereafter referred to as ACE2ecd), the RBD (residues 319−541) of WT, Alpha, Beta, Gamma, Delta, Omicron (including B.1, BQ.1 and XBB subvariants) SARS‐CoV‐2, as well as the RBD of SARS‐CoV Spike (residues 306−527) were each cloned into a modified pFastBac vector with an N‐terminal gp67 signaling peptide and a C‐terminal thrombin cleavage site followed by a strep tag and a 6xHis tag. The RBD of HCoV‐NL63 (residues 481−616) was fused with an N‐terminal gp67 signaling peptide and a C‐terminal 6xHis tag, and cloned into a pFastBac vector. All other ACE2ecd isoforms were then constructed through site‐directed mutagenesis and verified by Sanger sequencing.

The sequences of the oligos are listed in Table S1.

### 4.3 Protein expression and purification

Recombinant bacmids were prepared using the BacToBac system (Invitrogen) following manufacturer's manual. Baculoviruses were generated by transfecting Gibco Sf9 cells (11496015, Thermo Fisher Scientific) at 70% confluency with freshly prepared bacmids using FuGene HD (Promega). After 72–96 h, the medium of transfected cells was harvested and used as P1 virus stocks. P2 viruses were generated from P1 stocks, supplemented with 2% FBS and stored at 4°C in the dark until further use.

For expression of the ACE2ecd, 1 L HighFive cells at 1.5–2 × 10^6^ cells/mL were infected with 20 mL P2 virus and harvested at 60 h by centrifugation at 3000 *g* for 10 min and then 15,000 *g* for 30 min. Phenylmethanesulfonyl fluoride (1 mM) was added in the supernatant and loaded onto an Excel Ni‐NTA column (GE Healthcare) equilibrated with buffer A (50 mM Tris–HCl, pH 8.0, 250 mM NaCl). The column was then washed with buffer A supplemented with 5 mM imidazole before being eluted with a linear gradient of 5−500 mM imidazole. After SDS‐PAGE examination, elution fractions containing the ACE2ecd protein were pooled and further purified via a Superdex S200 column (GE Healthcare) in buffer B (20 mM Tris–HCl, pH 8.0, 150 mM NaCl) on SEC fractions containing ACE2ecd were then pooled, aliquoted and stored at −80°C. Other ACE2ecd isoforms and different RBD proteins were expressed and purified using similar methods as described before.

### 4.4 Transfection

The ACE2‐KO cell line was established by transfection of WT 293FT cells. The Cas9 expression vector pCMV‐Cas9 and sgACE2‐KO expression plasmid at a mass ratio of 3:2 were mixed with transfection reagent Lipofectamine LTX Reagent (Life, Invitrogen) in a six‐well plate. After 72 h, the transfected cells were seeded in 96‐well plate at a density of one cell per well. And after 3−4 weeks, the genomic DNA was extracted from the cells by using QuickExtract DNA Extraction Solution (QE09050, Epicentre) or the cells were harvested for western blotting to confirm the knockout of *ACE2*.

For generation of EF1αP‐KI cells, WT 293FT cells were transfected with Cas9 expression vector pCMV‐Cas9, sgACE2‐KI, and EF1αP donor plasmid at a mass ratio of 3:2:3 and mixed with transfection reagent Lipofectamine LTX Reagent (Life, Invitrogen) in a six‐well plate. After 72 h, the transfected cells were seeded in 96‐well plate at a density of one cell per well. And After 3−4 weeks, the genomic DNA was extracted and the cells were harvested for western blotting to confirm the knocking of EF1α promoter.

To exogenously overexpress WT or isoform ACE2, ACE2‐KO 293FT cells were transfected with WT ACE2 expression plasmid (or ACE2‐K353del, ACE2‐AAA/K353del, or ACE2‐AKA/K353del expression plasmid) in a six‐well plate. After 72 h, the cells were harvested for western blotting, flow cytometry, or angiotensin II converting enzymatic activity assay.

For prime editing of *ACE2*, EF1αP‐KI 293FT cells were transfected with 1 μg PE2 expression vector pCMV‐PE2 (Addgene, 132775), 0.44 μg pegRNA expression plasmid, and 0.2 μg nicking sgRNA expression plasmid in a 24‐well plate at a density of 1.6 × 10^5^ per well. After 72 h, the transfected cells were harvested for genomic DNA extraction, western blotting, or angiotensin II converting enzymatic assay.

### 4.5 DNA library preparation and sequencing

Target genomic sequences were amplified using Phanta Max Super‐Fidelity DNA Polymerase (P505, Vazyme) and primer sets flanking target sites. Indexed DNA libraries were prepared and sequenced as previously reported.^18^ The pegRNA target sequences and polymerase chain reaction (PCR) primers are listed in Table S1.

### 4.6 ACE2 codon substitution and deletion frequency calculation

Frequencies of amino acid substitutions were calculated at the sites with at least 1000 independent reads mapped, and obvious amino acid substitutions were only detected at the targeted editing sites. Amino acid substitution frequencies were evaluated by sequenced reads without indels and calculated as previously described.^18^ Counts of reads with desired mutations and total reads at mapped sites are listed in Table S2.

Intended indel frequencies were calculated as follows: (count of reads with only intended indel at the target site)/(count of total reads covering the target site). The edited reads containing intended indels were also requested not to carry any other point mutations or indels. These counts are also listed in Table S2.

### 4.7 OT indel frequency calculation

Potential pegRNA‐dependent OT sites were predicted by Cas‐OFFinder allowing up to four mismatches.^32^ Indel frequencies for pegRNA‐dependent OT sites were calculated according to reported CFBI pipeline (https://github.com/YangLab/CFBI, v1.0.0). These counts are listed in Table S3.

### 4.8 Antibodies

Antibodies were purchased from the following sources: mouse monoclonal antibody to β‐actin (Absci, AB21800), rabbit monoclonal antibody to ACE2 (Abcam, ab108252), and phycoerythrin‐conjugated anti‐6X His tag antibody (Abcam, ab72467).

### 4.9 Western blotting

Transfected cells were lysed in NP40 lysis buffer for 30 min on ice, and the total protein levels of cell lysate were assessed by BCA kit (Bicinchoninic acid assay kit, Thermo Fisher Scientific). The cell lysates were separated by SDS‐PAGE (Genscript) and proteins were transferred to nitrocellulose membranes (Thermo Fisher Scientific). After blocking with TBST containing 5% (w/v) nonfat dry milk and 1% BSA (Bovine Serum Albumin) for 2 h at room temperature. The primary antibody was added to the membrane overnight. After washing three times, the membranes were reacted with HRP (Horseradish Peroxidase)‐conjugated secondary antibodies for 1 h. Amplified protein markings were identified in ECL (Enhanced Chemiluminescence, Thermo Fisher Scientific) and detected with Amersham Imager 680.

### 4.10 Angiotensin II converting enzymatic assay

For recombinant ACE2ecd or its edited isoforms, the enzymatic assay was performed using the ACE2 Protease Activity Assay kit (Biovision). Briefly, different concentrations of substrate were prepared in a 96‐well plate with a total reaction volume of 100 μL at 25°C. The final concentration of ACE2ecd or its edited isoforms was 1 μM, and the fluorescence signals were measured (Ex/Em 320 nm/420 nm) in a kinetic mode on MD‐SpectraMax i3 (Molecular Devices). The data were analyzed by Michaelis–Menten curve fitting with Origin software (OriginLab). For enzymatic analysis of cell lysates, transfected cells were lysed with lysis buffer and assayed according to the manual of ACE2 Activity Assay Kit (AssayGenie, BN01071). BCA Protein Assay Kit (YEASEN, 20202ES76) was used to detect the protein concentration in the lysate. Fluorescence data were measured with MD‐SpectraMax i3 (Molecular Devices) and fitted as described above. The enzyme activity counts are listed in Table S4.

### 4.11 Surface plasmon resonance assay

SPR experiments were performed using a Biacore 8K instrument (GE Healthcare). All assays were performed in HBS‐EP+ buffer (Cytiva) at 25°C. For affinity determinations, recombinant RBD proteins of different SARS‐CoV‐2 strains and that of HCoV‐NL63 or SARS‐CoV were each immobilized on CM5 sensor chips (GE Healthcare). Serial dilutions (from 200 to 3.125 nM) of recombinant WT or isoform ACE2ecd were injected over both ligand and reference flow cells. Data were fitted to a 1:1 Langmuir‐binding model using the Biacore Insight Evaluation Software (GE Healthcare).

### 4.12 Homology model building of selected ACE2 isoforms

Models of the ACE2ecd isoforms, namely AAA (Q24A/D30A/K31A), AKA (Q24A/D30K/K31A), K353del, AAA/K353del, and AKA/K353del, were derived by homology modeling using SWISS‐MODEL.^51^

### 4.13 Flow cytometry

Recombinant RBD proteins of WT, Alpha, Beta, Gamma, Delta, and Omicron (including B.1, BQ.1, and XBB) SARS‐CoV‐2 strains and that of HCoV‐NL63 or SARS‐CoV were incubated at a concentration of 20 μg/mL with 1 × 10^6^ ACE2‐KO 293FT cells or ACE2‐KO 293FT cells that exogenously overexpress WT or ACE2 isoform in 500 μL phosphate buffered saline (PBS) for 60 min at room temperature. After washing twice with PBS containing 2% FBS (16000‐044, Gibco), cells were resuspended and incubated with phycoerythrin‐conjugated anti‐His antibody (Abcam, ab72467) for 30 min at 4°C in dark. And then, the cells were washed three times and resuspended in PBS containing 2% FBS before being analyzed using CytoFLEX (Beckman Coulter). ACE2‐KO 293FT cells served as negative controls. Data were analyzed using FlowJo software.

### 4.14 Packaging of pseudoviruses

PsVs bearing coronavirus spike proteins were generated by co‐transfecting HEK293T with Luc‐transfer vector (encoding luciferase reporter gene), psPAX2 packaging plasmid (encoding a defective HIV‐1 genome), and pMD2.G plasmid (encoding either the SARS‐CoV spike protein or C‐terminal 19 AA truncated SARS‐CoV‐2 spike protein or C‐terminal 18 amino acid truncated HCoV‐NL63 spike protein). After overnight incubation, the cells were washed twice with PBS and changed to fresh DMEM medium. After 48 h of transfection, the supernatant was harvested and centrifuged at 1000 *g* for 5 min, collected, concentrated, and stored at −80°C until further use.

### 4.15 Analysis of pseudovirus entry

Pseudotyped SARS‐CoV, HCoV‐NL63, and SARS‐CoV‐2 Delta strains were packed in house as described above. PsVs of other SARS‐CoV‐2 strains were purchased from the following sources: SARS‐CoV‐2‐Fluc WT (Vazyme, DD1402‐03), SARS‐CoV‐2‐Fluc Alpha (Vazyme, DD1440‐03), SARS‐CoV‐2‐Fluc Beta (Vazyme, DD1441‐03), SARS‐CoV‐2‐Fluc Gamma (Vazyme, DD1446‐03), and SARS‐CoV‐2‐Fluc Omicron (Vazyme, DD1754‐03). The PsVs were used to challenge ACE2‐KO 293FT cells (1.5 × 10^4^ per well in 96‐well plate) that exogenously overexpressed ACE2 isoforms or PE3‐edited EF1αP‐KI 293FT cells. After 48 h, challenged cells were washed twice with PBS, lysed and analyzed for intracellular luciferase activity with the Bright‐Lite Luciferase Assay System (Vazyme, DD1204). Luminescence was recorded with Tecan‐Spark (Tecan). Luminescence was recorded with Tecan‐Spark (Tecan) and is listed in Table S5.

### 4.16 Live SARS‐CoV‐2 infection

Live SARS‐CoV‐2 infection was performed using a clinical isolate (Delta, B.1.617.2) in a certified Biosafety level 3 laboratory (BSL3) at Shanghai Medical College of Fudan University. PE3‐edited EF1αP‐KI 293FT cells were infected with live virus at an multiplicity of infection of 0.01 in six‐well plates (1 × 10^6^ per well) and incubated at 37°C. Twenty‐four hours post‐infection, cell culture supernatants were treated with TRIzol LS Reagent (Thermo Fisher Scientific) and total RNA was extracted. Viral genomic RNA copies in the supernatants were quantified through RT‐PCR using One‐Step PrimeScript RT‐PCR Kit (Takara) with primer sets (SARS‐CoV‐2‐N‐F, SARS‐CoV‐2‐N‐R, and SARS‐CoV‐2‐N‐probe, sequences listed in Table S1). A well with no cell was similarly calculated with live virus to serve as negative control and viral RNA copies in this well were used for background subtraction. The relative copy numbers of Delta live virus genome are listed in Table S6.

## AUTHOR CONTRIBUTIONS

B.Y., J.C., L.Y., and L.L. conceived, designed, and supervised the project. W.Z., J.L., and X.W. performed most experiments with the help of J.X. on homology model building, Y. H., J.W., and Q.Q. on plasmid construction, cell culture, and transfection. W.X. performed live virus‐related experiments under the supervision of L.L. J.W. prepared libraries for DNA sequencing and B.Q.G. performed bioinformatics analyses supervised by L.Y. W.L. and X.Y. provided technical support. B.Y., J.C., L.Y., and L.L. wrote the paper with inputs from the other authors. B.Y. managed the project. All authors have read and approved the final manuscript.

## CONFLICT OF INTEREST STATEMENT

The authors declare they have no conflicts of interest.

## ETHICS STATEMENT

Not applicable.

## Supporting information



Supporting information

Supporting information

Supporting information

Supporting information

Supporting information

Supporting information

Supporting information

## Data Availability

The deep sequencing data generated in this study are available at the NCBI Sequence Read Archive database under PRJNA999436.
