# Supplementary material for: Prime editor‐mediated functional reshaping of ACE2 prevents the entry of multiple human coronaviruses, including SARS‐CoV‐2 variants
Source: MedComm (2020). 2023 Sep 10;4(5):e356. doi: 10.1002/mco2.356 (PMC10492923; doi:10.1002/mco2.356)
Supplement: Supplementary file 1 — Supporting information [file MCO2-4-e356-s007.pdf]

Supporting Information for

Prime editor-mediated functional reshaping of *ACE2* prevents the entry of SARS-CoV-2 variants-included HCoVs

Wenwen Zhao[1,2,3,4,5,#], Jifang Li[1,2,3,4,5,#], Xiao Wang[1,2,4,#], Wei Xu[6,#], Bao-Qing Gao[7,#], Jiangchao Xiang[1,3,4,5], Wei Liu[8], Jing Wu[1,2], Qilian Qi[1], Jia Wei[9], Xiaoyu Yang[8], Lu Lu[6,*], Li Yang[9,*], Jia Chen[1,2,3,4,*] & Bei Yang[1,3,*]

Correspondence to:
Bei Yang (yangbei@shanghaitech.edu.cn)
Jia Chen (chenjia@shanghaitech.edu.cn)
Li Yang (liyang_fudan@fudan.edu.cn)
Lu Lu (lul@fudan.edu.cn)

This PDF file includes:
Figures S1 to S7
Table S1

ACE2 resi.	RBD resi.	interaction	6m17	7kj4
**Q24**	**N487**	**sidechain to sidechain H bond**	**yes**	**yes**
T27	Y473	sidechain to sidechain hydrophobic stacking	no	yes
**M82**	**F486**	**sidechain to sidechain hydrophobic stacking**	**yes**	**yes**
**Y83**	**F486**	**sidechain to sidechain hydrophobic stacking**	**yes**	**yes**
Y83	N487	sidechain to sidechain H bond	yes	no
Y83	Y489	sidechain to sidechain H bond	no	yes
**D30**	**K417**	**sidechain to sidechain H bond**	**yes**	**yes**
**K31**	**Y489**	**sidechain to sidechain hydrophobic stacking**	**yes**	**yes**
**K31**	**Q493**	**sidechain to sidechain H bond**	**yes**	**yes**
**H34**	**Y453**	**sidechain to sidechain hydrophobic stacking**	**yes**	**yes**
**H34**	**Y453**	**sidechain to sidechain H bond**	**yes**	**yes**
E35	Q493	sidechain to sidechain H bond	no	yes
E37	Y505	sidechain to sidechain H bond	yes	no
**D38**	**Y449**	**sidechain to sidechain H bond**	**yes**	**yes**
**Y41**	**T500**	**sidechain to mainchain H bond**	**yes**	**yes**
**Y41**	**T500**	**sidechain to sidechain H bond**	**weak**	**yes**
Y41	N501	sidechain to sidechain H bond	yes	no
**Q42**	**Y449**	**sidechain to sidechain H bond**	**yes**	**weak**
Q42	Q498	sidechain to sidechain H bond	yes	no
**K353**	**G496**	**sidechain to mainchain H bond**	**yes**	**yes**
K353	Q498	sidechain to sidechain H bond	no	yes
K353	N501	sidechain to sidechain H bond	yes	no
**K353**	**G502**	**mainchain to mainchain H bond**	**yes**	**yes**
**K353**	**Y505**	**sidechain to sidechain hydrophobic stacking**	**yes**	**yes**
D355	T500	sidechain to sidechain H bond	yes	no
**D355**	**T500**	**sidechain to mainchain H bond**	**yes**	**yes**
R357	T500	sidechain to sidechain H bond	yes	no

Figure S1 Consensus interface residues between ACE2 and SARS-CoV-2 RBD.

Only ACE2 residues that bind SARS-CoV-2 RBD in both structures are considered crucial and chosen for editing (bold). Interface residues are colored according to their attributions to the three clusters illustrated in Fig 1A close-up views.

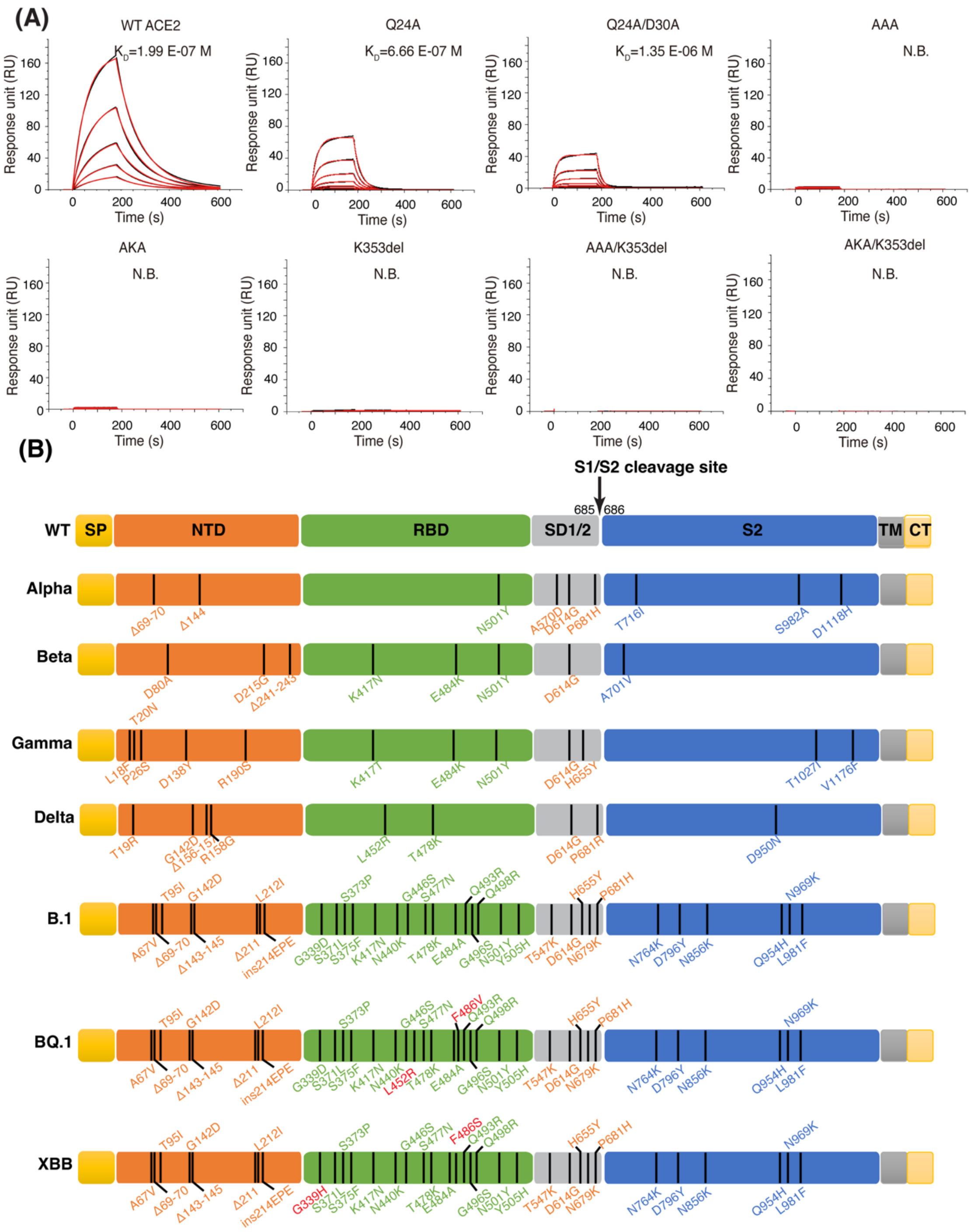

Figure S2 SPR sensorgrams and Spike mutations mapping of SARS-CoV-2 VOCs.

(A) Binding curves of WT ACE2ecd and its edited isoforms to immobilized RBD from SARS-CoV-2 WT strain. Data are shown as black lines and the best fit of the

data to a 1:1 binding model are shown in red. **(B)** Mutation mapping on the Spike proteins from wildtype SARS-CoV-2 and indicated variants.

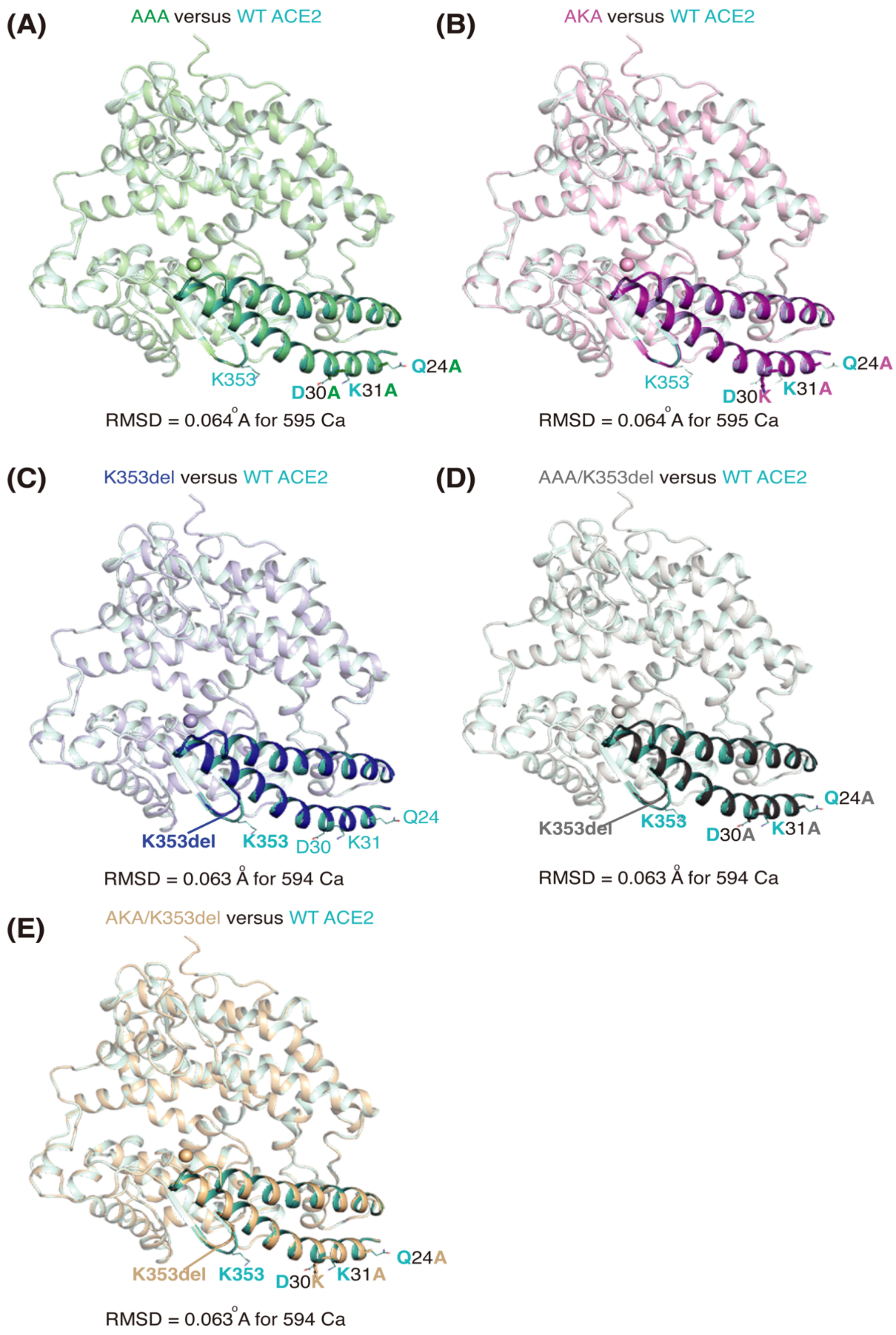

Figure S3 Ectodomain structures of edited ACE2 isoforms remain largely unchanged.

(A-E) Homology models of edited ACE2 isoforms AAA (A), AKA (B), K353del (C), AAA/K353del (D) and AKA/K353del (E) are superimposed onto the representative structure of WT ACE2ecd (PDBid: 6m17) and corresponding RMSDs for Cα atoms are shown. The interface mainly involves helices α1-α2 and the loop between β3 and β4 strands on ACE2, cartoon representations of these structural motifs are highlighted while the rest are rendered transparent for clarity. Edited interface residues are shown as stick models in the structures of edited isoforms and line models in WT ACE2. Editing of Q24, D30 and K31 only affects the sidechains of corresponding residues, and K353 deletion shortens the loop between β3 and β4 strands. No large conformational changes of ACE2 are induced by any of these edited residues or their combinations.

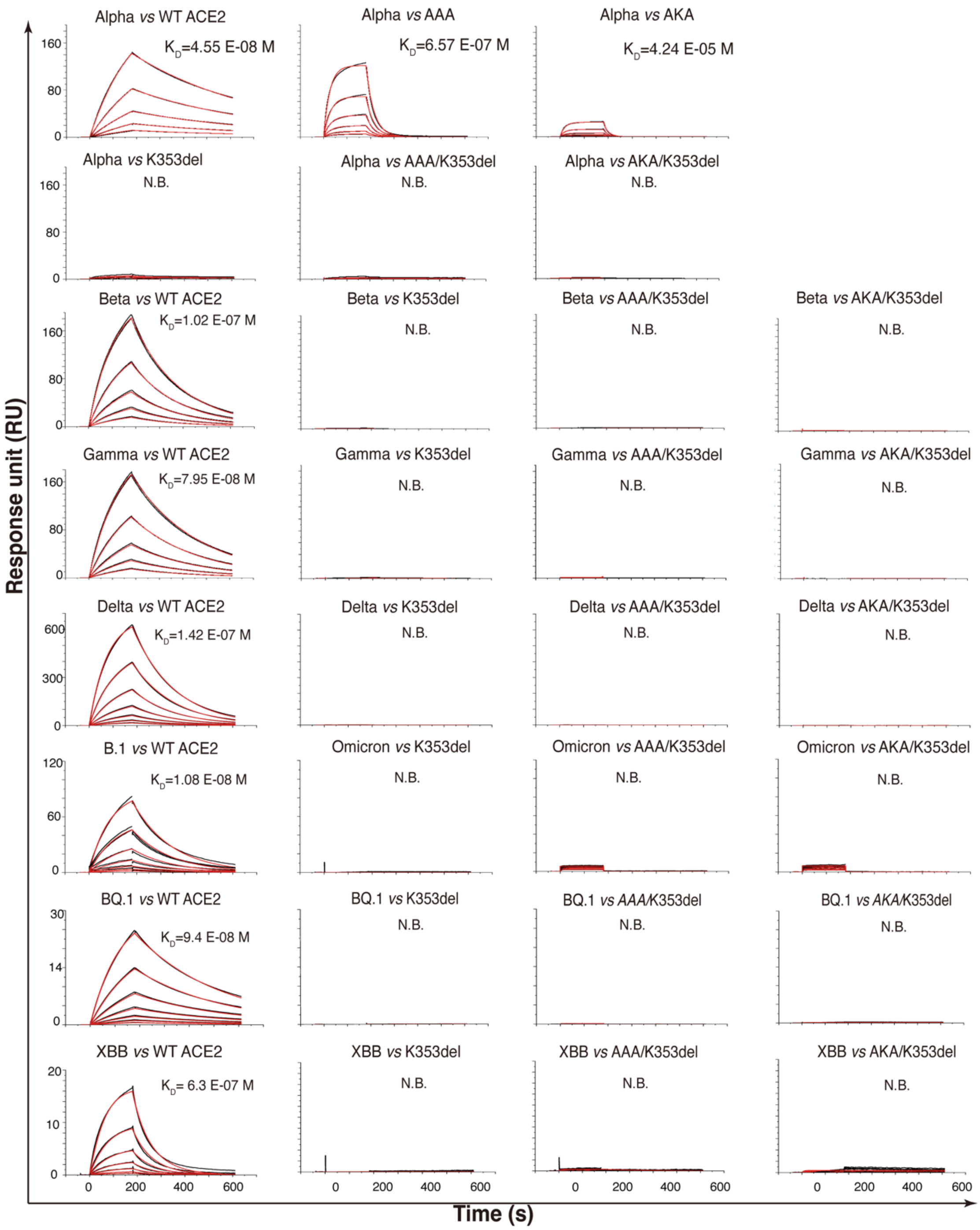

Fiugre S4 SPR sensorgrams.

Binding curves of WT ACE2 and its edited isoforms to immobilized RBD from SARS-CoV-2 alpha, beta, gamma, delta, Omicron B.1, Omicron BQ.1 and Omicron XBB strains. Data are shown as black lines and the best fit of the data to a 1:1 binding model are shown in red.

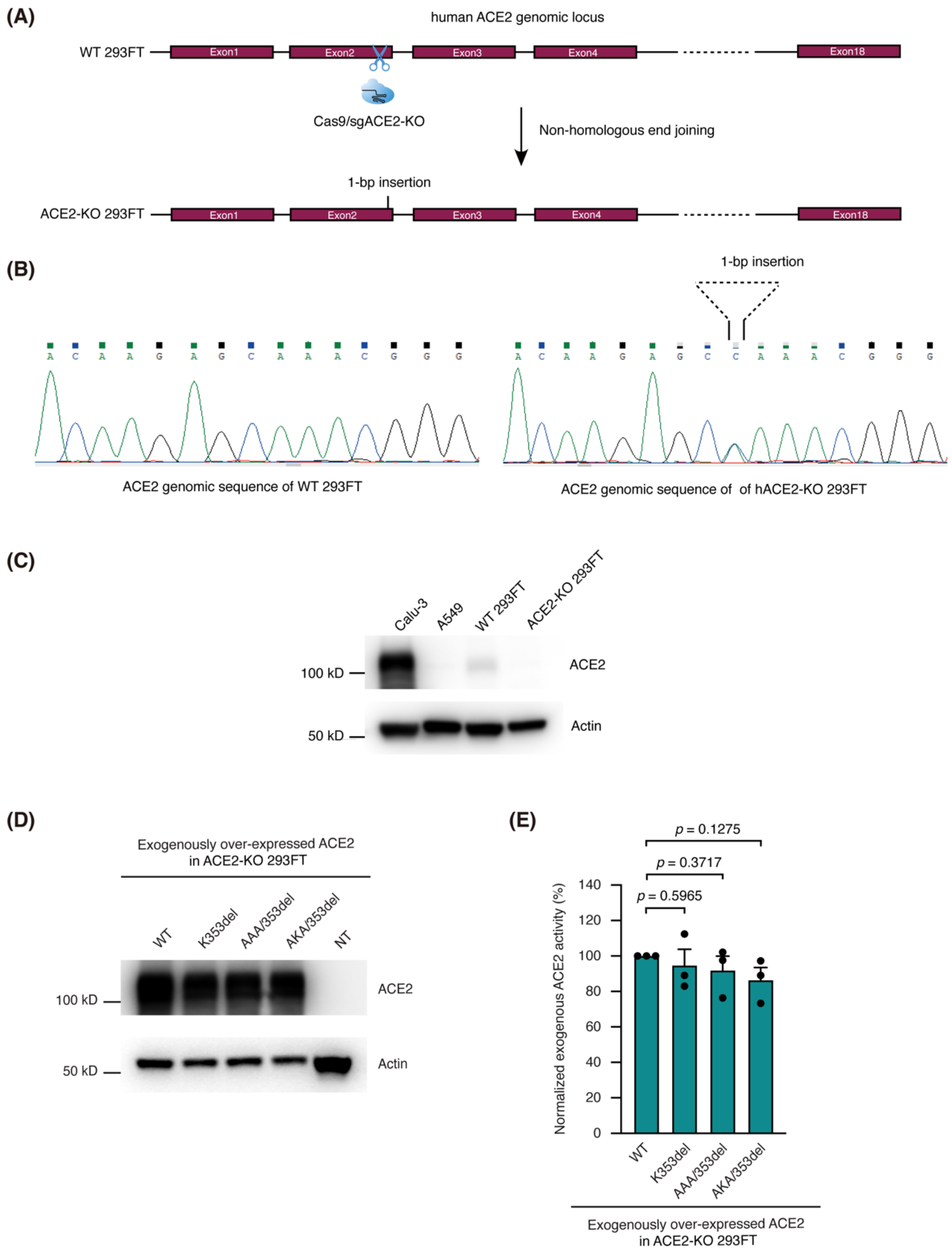

Figure S5 Generation of the ACE2-KO 293FT cells.

(A) Schematic overview depicting the generation of the ACE2-KO 293FT cells wherein the expression of endogenous ACE2 was abolished. Briefly, indels were introduced into the genome of WT 293FT cells at indicated site through

Cas9/sgACE2-KO triggered non-homologous end joining. The final stable cell line contains 1-bp insertion in the exon2 of *ACE2* gene. **(B)** Sanger sequencing results of the edited region in ACE2-KO 293FT cells. **(C)** Western blots of ACE2 were performed for Calu-3, A549, WT 293FT and ACE2-KO 293FT cells, noting that the expression of ACE2 was eliminated in ACE2-KO 293FT cells. **(D-E)** Western blots of ACE2 proteins (D) and Ang II converting activity measurements (E) were performed for non-transfected ACE2-KO 293FT cells (NT) and ACE2-KO 293FT cells overexpressing WT ACE2 (WT) or its edited isforms (K353del, AAA/K353del or AKA/K353del). **(D)** Data shown are representative results for three experiments. **(E)** The Ang II converting activity of ACE2-KO 293FT cells exogenously overexpressing WT ACE2 was taken as 100%. Data are shown as means + SD (n=3). *P* values are from *t* test.

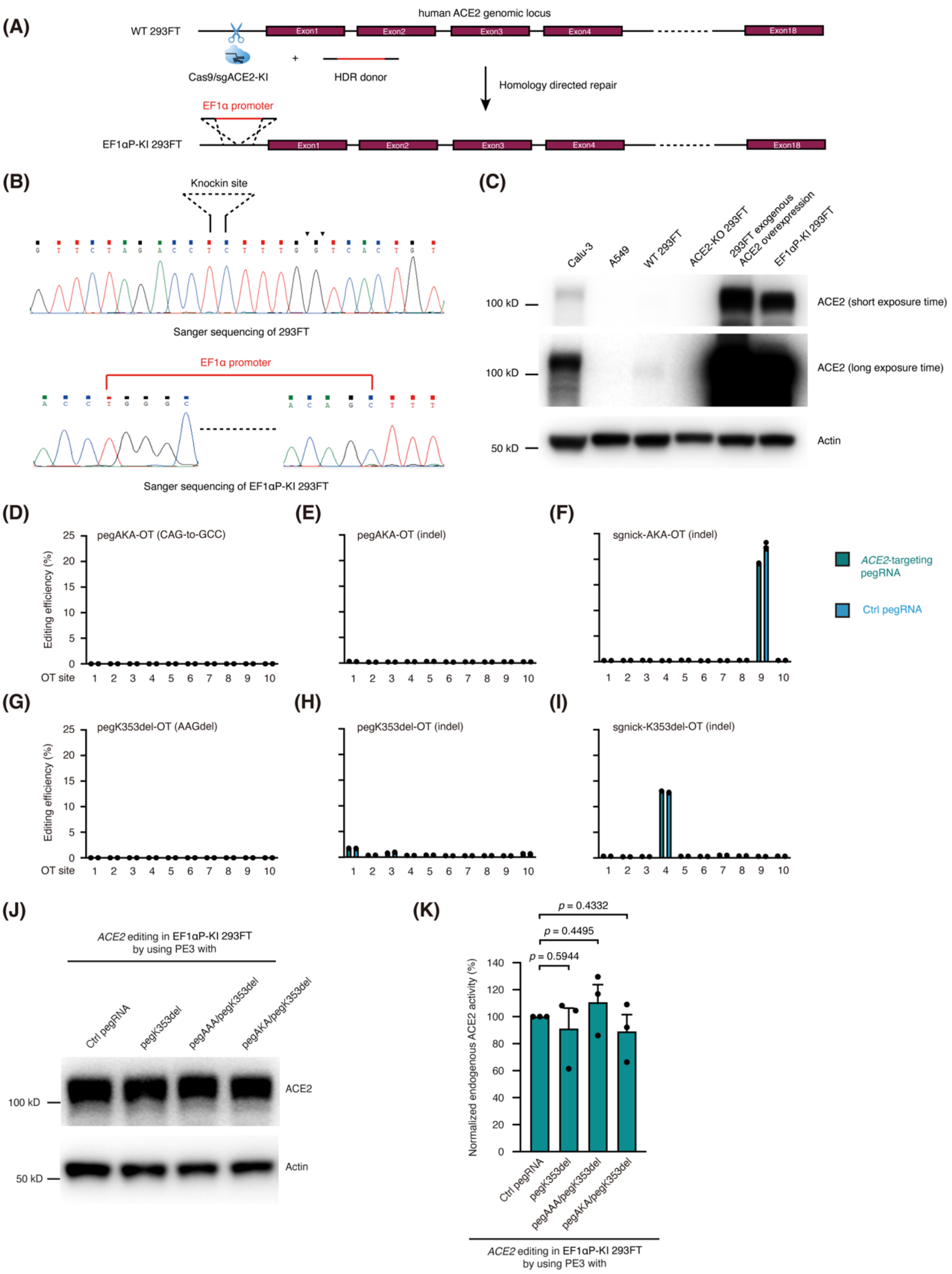

Figure S6 Generation of EF1αP-KI 293FT cells and editing of endogenous *ACE2*.

(A) Schematic overview depicting the strategy used to generate the EF1αP-KI 293FT cells which stably express endogenous ACE2 at a high level. Briefly, homology directed repair (HDR) was triggered by Cas9/sgACE2-KI at indicated sites in the

presence of a HDR donor to insert a EF1α promoter into the *ACE2* promoter region. **(B)** Sanger sequencing results of ACE2 promoter region in EF1αP-KI 293FT cells. **(C)** Western blots of ACE2 were performed for Calu-3, A549, WT 293FT, ACE2-KO 293FT, ACE2-KO 293FT exogenously overexpressing WT ACE2 and EF1αP-KI 293FT cells. **(D-E)** Frequencies of PE-mediated amino acid substitutions (D) or indels (E) at top 10 predicted off-target sites based on the spacer sequence of pegRNA for generating ACE2-AKA. Data are shown as means + SD (n=3). **(F)** Frequencies of PE-mediated indels at top 10 predicted off-target sites based on the spacer sequence of nicking sgRNA for generating ACE2-AKA. **(G-H)** Frequencies of PE-mediated intended editing (G) or indels (H) at top 10 predicted off-target sites based on the spacer sequence of pegRNA for generating ACE2-K353del. **(I)** Frequencies of PE-mediated indels at top 10 predicted off-target sites based on the spacer sequence of nicking sgRNA for generating ACE2-K353del. **(J-K)** The ACE2 expression levels (J) and Ang II converting activities (K) were similar among EF1αP-KI 293FT cells edited with indicated pegRNAs. **(J)** Data shown are representative results for three experiments. **(K)** The Angiotensin II Converting activities of mock-edited EF1αP-KI 293FT cells was taken as 100%. Data are shown as means + SD (n=3). *P* values are from *t* test.

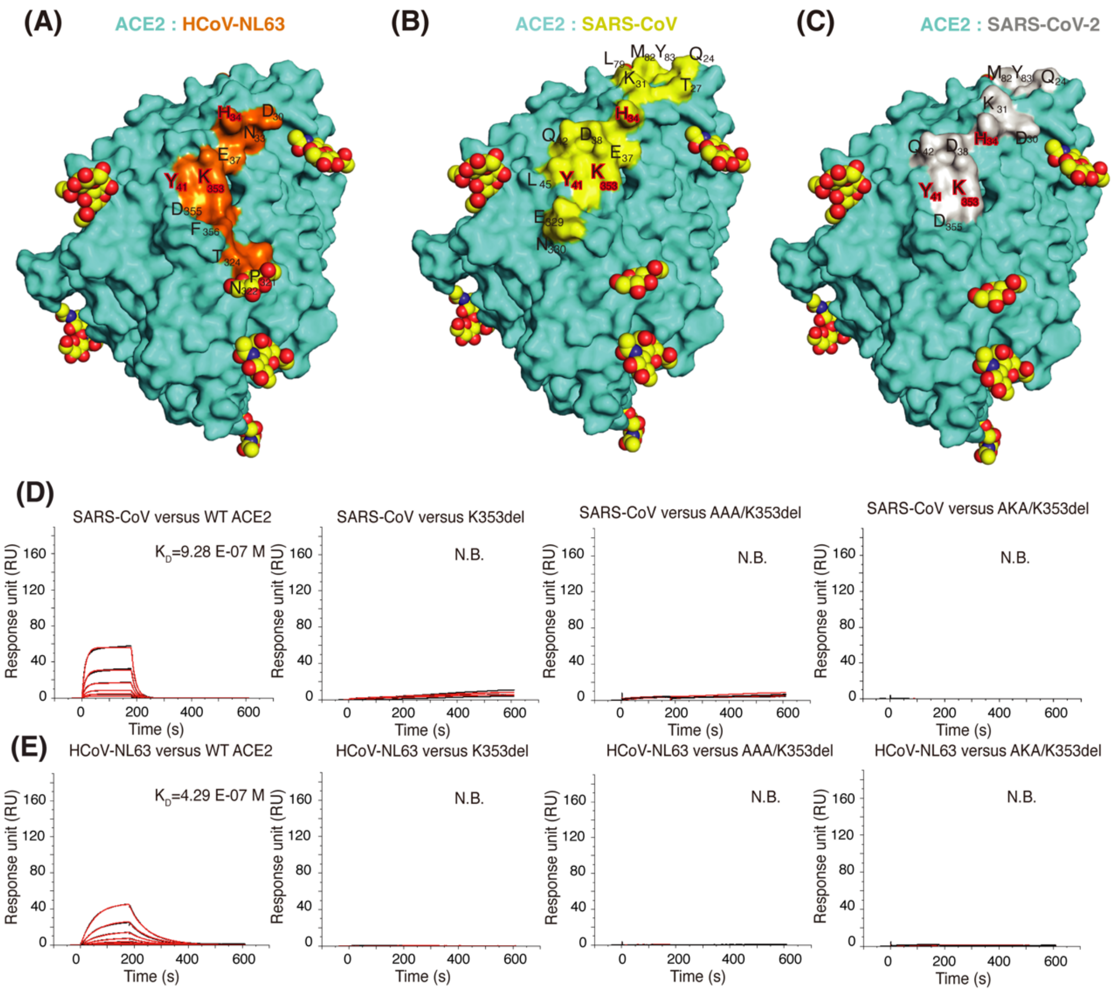

Figure S7 Selected mutations render ACE2 resistant to the binding of RBDs from SARS-CoV and HCoV-NL63.

(A-C) Footprint of RBDs from (A) HCoV-NL63 (orange), (B) SARS-CoV (yellow) or (C) SARS-CoV-2 (grey) on the surface presentation of ACE2 (cyan). ACE2 residZues that makes direct contacts with the RBDs from all three HCoVs, *i.e.,* H34, Y41 and K353, are highlighted with black labels in red frames. N-glycans on ACE2 are shown as spheres. **(D-E)** SPR binding curves of the ectodomains of WT ACE2 or its edited isoforms to immobilized RBDs from SARS-CoV (D) or HCoV-NL63 (E). Data are shown as black lines and the best fit of the data to a 1:1 binding model are shown in red.

Table S1 Oligos used for plasmid construction and pegRNA/sgRNA target sequences, PCR primers for amplifying genomic DNA and for quantifying viral genomic RNA copies.

Primer name	Sequence (5' to 3')
ACE2_PCR_F	AGGGAGACCCAAGCTGGCTAGACTCGAGCGGCC GCATGTCAAGCTCTTCCTGGCT
ACE2_PCR_R	TCGTATGGGTAAGAACCACCAGAGGATCCAAAG GAGGTCTGAACATCATCAG
ACE2_AAA_PCR_F	GCGGCCAAGACATTTTTGGCCGCGTTTAACCACG AAGCCGAAGACCTGT
ACE2_AAA_PCR_R	AACGCGGCCAAAAATGTCTTGGCCGCTTCCTCAA TGGTGGACTGAGCAGCA
ACE2_AKA_PCR_F	GCGGCCAAGACATTTTTGAAGGCGTTTAACCACG AAGCCGAAGACCTGT
ACE2_AKA_PCR_R	CGCCTTCAAAAATGTCTTGGCCGCTTCCTCAATG GTGGACTGAGCA
ACE2_K353del_PCR_F	GACCTGGGGGGCGACTTCAGGATCCTTATGTGCA CAAA
ACE2_K353del_PCR_R	TGAAGTCGCCCCCCAGGTCCCAAGCTGTGGGA
sgACE2_KO	TCTCAGAAGACAAGAGCAAACGG
sgACE2_KO_FOR	ACCGTCTCAGAAGACAAGAGCAAA
sgACE2_KO_REV	AAATTTTGCTCTTGTCTTCTGAGA
sgACE2_KI	TCCCTAGTTCTAGACCTCTTTGG
sgACE2_KI_FOR	ACCGTCCCTAGTTCTAGACCTCTT
sgACE2_KI_REV	AAATAAGAGGTCTAGAACTAGGGA
KI_donor_F1	GTGAAGAAGCGATTATGATCTCTCCTCTAGACTG ATCAGAAATATGAATTCC
KI_donor_R1	GATGTGCGCTCTGCCCAGGTCTAGAACTAGGGAT CAT
KI_donor_F2	TAGACCTGGGCAGAGCGCACATCGCCCACAGT
KI_donor_R2	TTACAGTGACCAAAGCTGTGTTCTGGCGGCAAAC CC
KI_donor_F3	AACACAGCTTTGGTCACTGTAAAATTATAACATT TTCCGTG
KI_donor_R3	CCCAGGCTCCCAGGCCCTGAACGCGTACAGATAT GTAACAGATTTTAG
sgNick_Q24	GACATTCTCTTCAGTAATATTGG
sgNick_Q24_FOR	ACCGGACATTCTCTTCAGTAATAT
sgNick_Q24_REV	AAATATATTACTGAAGAGAATGTC
sgNick_M82	AGAATAATGCTGGGGACAAATGG

sgNick_M82_FOR	ACCGAGAATAATGCTGGGGACAAA
sgNick_M82_REV	AAATTTTGTCCCCAGCATTATTCT
sgNick_K353	ATTTCCTGGGTCCGTTAGCATGG
sgNick_K353_FOR	ACCGATTTCCTGGGTCCGTTAGCA
sgNick_K353_REV	AAATTGCTAACGGACCCAGGAAAT
U6_peg_F	AGGTACGGGAGGTACTTGGAGCGGCCGCAATAAAATATCT
U6_Q24A_F	GTCAGTCCACCATTGAGGAACGTTTTAGAGCTAGAAATAGCAA
U6_Q24A_R	GTTCCTCAATGGTGGACTGACGGTGTTTCGTCCTTTCCACAAGA
U6_Q24A_R1	AGTCCACCATTGAGGAAGCCGCCAAGACATTTTTGGAGGACCGACTCGGTCCCACTT
U6_Q24A_R2	ATTTGTCTCGAGGTCGAGAATTCTTTAAAAAAAAGTCCACCATTGAGGAAGCC
U6_D30A_F	GGGAACAGGCCAAGACATTTTGTTTTAGAGCTAGAAATAGCAA
U6_D30A_R	AAAATGTCTTGGCCTGTTCCCGGTGTTTCGTCCTTTCCACAAGA
U6_D30A_R1	AACAGGCCAAGACATTTTTGGCAAAGTTTAACCACGAGGACCGACTCGGTCCCACTT
U6_D30A_R2	ATTTGTCTCGAGGTCGAGAATTCTTTAAAAAAAAACAGGCCAAGACATTTTTGG
U6_D30K_R1	AACAGGCCAAGACATTTTTGAAGAAGTTTAACCACGAGGACCGACTCGGTCCCACTT
U6_D30K_R2	CATTTGTCTCGAGGTCGAGAATTCTTTAAAAAAAAACAGGCCAAGACATTTTTGA
U6_K31A_F	CGAGAACAGGTCTTCGGCTTCGGTTTTAGAGCTAGAAATAGCAA
U6_K31A_R	CGAAGCCGAAGACCTGTTCTCGGTGTTTCGTCCTTTCCACAAGA
U6_K31A_R1	AACAGGTCTTCGGCTTCGTGGTTAAAGGCGTCCAAAAATGTCTTGGACCGACTCGGTCCCACTT
U6_K31A_R2	TTTGTCTCGAGGTCGAGAATTCTTTAAAAAAAAACAGGTCTTCGGCTTCGTGGTTAAAG
U6_H34A_F	CGAGAACAGGTCTTCGGCTTCGGTTTTAGAGCTAGAAATAGCAA
U6_H34A_R	CGAAGCCGAAGACCTGTTCTCGGTGTTTCGTCCTTTCCACAAGA
U6_H34A_R1	AAAAACAGGTCTTCGGCTTCTGCGTTAAACTTGTCCAGGACCGACTCGGTCCCACTT
U6_H34A_R2	CCATTTGTCTCGAGGTCGAGAATTCTTTAAAAAAAAACAGGTCTTCGGCTTCTG

U6_D38A_R1	AAGTTTAACCACGAAGCCGAAGCCCTGTTCTATCAAAGTGGACCGACTCGGTCCCACTT
U6_D38A_R2	AGTCCACCATTGAGGAACAGGCCAAGACATTTTTGGACAAGTTTAACCACGAAGCCGAA
U6_D38A_R3	CATTTGTCTCGAGGTCGAGAATTCTTTAAAAAAAAGTCCACCATTGAGGAACAGGCCA
U6_Y41A_R1	ACGAAGCCGAAGACCTGTTCGCTCAAAGTTCACTTGCTTGGACCGACTCGGTCCCACTT
U6_Y41A_R2	TTGAGGAACAGGCCAAGACATTTTTGGACAAGTTTAACCACGAAGCCGAAGACCTGTTC
U6_Y41A_R3	ATTTGTCTCGAGGTCGAGAATTCTTTAAAAAAAAGTCCACCATTGAGGAACAGGCC
U6_Q42A_R1	ACGAAGCCGAAGACCTGTTCTATGCCAGTTCACTTGCTTGGACCGACTCGGTCCCACTT
U6_M82S_F	CGAGATTCTGAATTTCTTGTAGGTTTTAGAGCTAGAAATAGCAA
U6_M82S_R	CTACAAGAAATTCAGAATCTCGGTGTTTCGTCCTTTCCACAAGA
U6_M82S_R1	GAATTTCTTGTAGTGGATAGCTTTGGGCAAGTGTGGACTGGACCGACTCGGTCCCACTT
U6_M82S_R2	TTTGTCTCGAGGTCGAGAATTCTTTAAAAAAAATTCTGAATTTCTTGTAGTGGATAGCT
U6_Y83A_R1	AATTTCTTGTAGTGGCGCCATTTGGGCAAGTGTGGAGGACCGACTCGGTCCCACTTTT
U6_Y83A_R2	TTTGTCTCGAGGTCGAGAATTCTTTAAAAAAAATTCTGAATTTCTTGTAGTGGCGCCAT
U6_K353A_F	CGACAGCTTGGGACCTGGGGAAGTTTTAGAGCTAGAAATAGCAA
U6_K353A_R	TTCCCCAGGTCCCAAGCTGTCGGTGTTTCGTCCTTTCCACAAGA
U6_K353A_R1	GGACCTGGGGGCAGGAGATTTCAGGTAGTGGGGCTGATAGGACCGACTCGGTCCCACTT
U6_K353A_R2	ATTTGTCTCGAGGTCGAGAATTCTTTAAAAAAAAGCTTGGGACCTGGGGGCAGGAGATT
U6_K353H_R1	AATCTCCTGCCCCCAGGTCCCAAGCTTTTTTTAAAGAATTCTCGACCTCGAGACAAAT
U6_K353H_R2	TTGTCTCGAGGTCGAGAATTCTTTAAAAAAAAGCTTGGGACCTGGGGCACGGAGATTTC
U6_K353del_R1	GGACCTGGGGGGAGATTTCAGGTAGTGGGGCTGATAGGACCGACTCGGTCCCACTT
U6_K353del_R2	ATTTGTCTCGAGGTCGAGAATTCTTTAAAAAAAAGCTTGGGACCTGGGGGGAGATTTCA
U6_D355A_R1	GACCTGGGGAAGGGCGCCTTCAGGTAGTGGGGCTGGACCGACTCGGTCCCACTTTTT

U6_D355A_R2	TTGTCTCGAGGTCGAGAATTCTTTAAAAAAAAGCTTGGGACCTGGGGAAGGGCGCCT
U6_AA_R1	GAAGCCGCCAAGACATTTTTGGCCAAGTTTAACCACGAAGGACCGACTCGGTCCCACTT
U6_AA_R2	ATTTGTCTCGAGGTCGAGAATTCTTTAAAAAAAAGTCCACCATTGAGGAAGCCGCCAAGACATTTTT
U6_AAA_R1	GAAGCCGCCAAGACATTTTTGGCCGCTTTTAACCACGAAGGACCGACTCGGTCCCACTT
U6_AAA_R2	AGTCCACCATTGAGGAAGCCGCCAAGACATTTTTGGCCGCTT
U6_AAA_R3	ATTTGTCTCGAGGTCGAGAATTCTTTAAAAAAAAGTCCACCATTGAGGAAGCCGC
U6_AKA_R1	GAAGCCGCCAAGACATTTTTGAAGGCCTTTAACCACGAAGGACCGACTCGGTCCCACTT
U6_AKA_R2	TTTAAAAAAAAGTCCACCATTGAGGAAGCCGCCAAGACATTTTTGAAG
ACE2_G1F	TGGCTCAGCAGATTGTTTAC
ACE2_G1R	GGGACTCCAAAATCAGGGATA
ACE2_G2F	TGGTCACTCTTAACCTAAACCT
ACE2_G2R	TACCTAGGCATAGAGAGAGA
ACE2_G3F	TACTCTCTGCTTCTACCAGTTCCA
ACE2_G3R	CAACGCCAATGGATGCATGATA
pegAKA-OT1	CCTGTTCCTCCGTGGTGGATTGA
pegAKA-OT1_G1F	AGACTCTCCACCCCCAGTAA
pegAKA-OT1_G1R	CCTGGGCTCCATCTCCTGTC
pegAKA-OT2	TCAAGTCCACCTTTGAGGATCTGG
pegAKA-OT2_G1F	GTACCTACCTCAGAAGACTG
pegAKA-OT2_G1R	CCAGACTCTAAGCCAGACTT
pegAKA-OT3	TCAGTCCCCCTTTGAGGAAGCTGG
pegAKA-OT3_G1F	CAAGAACCATAAAAGGCTCA
pegAKA-OT3_G1R	ATGTAGAGATAGCTGGTGGT
pegAKA-OT4	GCAGTGCACCATTGCGGAACTGG
pegAKA-OT4_G1F	GAACAGTGGATGTGCCTGTC
pegAKA-OT4_G1R	CTTCTCTGAAAGCCAGTTAG
pegAKA-OT5	TCAGTCCACCTCTGAGGATCAGG
pegAKA-OT5_G1F	CGGGCTTCTAGCCACCCAAA
pegAKA-OT5_G1R	ACTTGCTGGATGACCATGAT
pegAKA-OT6	TCAGTCCACCTGTAAGGAACGGG
pegAKA-OT6_G1F	TGGACGAAAGAGTGAGACTC
pegAKA-OT6_G1R	TGTTTTTGGTGACCTTGACA
pegAKA-OT7	TCAGTCCACCATGGAAGGACTGG
pegAKA-OT7_G1F	CTTGTGAGGCAAAGGTTGCA

pegAKA-OT7_G1R	AAGAAGGAAAGCAGGCTCTG
pegAKA-OT8	TCAGGACACCATGGAGGAACGGG
pegAKA-OT5_G1F	ATGTCTAGAGGATTCCCAGC
pegAKA-OT8_G1R	CCTCCTCACGAGATTAATGA
pegAKA-OT9	TGCAGTGCACCATTGCGGAACTGG
pegAKA-OT9_G1F	GAACAGTGGATGTGCCTGTC
pegAKA-OT9_G1R	CCAGTTAGACTGCGGCTGTT
pegAKA-OT10	TCAGTCCATCATTAAGGAATCCGG
pegAKA-OT10_G1F	GGTACTACTTACACCAAAGC
pegAKA-OT10_G1R	CTACGGATATAGCTTATGCA
sgNick_Q24_OT1	CAAGTAAATTTTGATAGGACTGG
sgNick_Q24_OT1_G1F	GGGCCAAAGTTGGCCTTACC
sgNick_Q24_OT1_G1R	CCTGGATGTCGCTGCTCAGA
sgNick_Q24_OT2	GAAGTGAACCTTTGATAGAAATGG
sgNick_Q24_OT2_G1F	CCTTGATCATAGGTCCTGGC
sgNick_Q24_OT2_G1R	CTGCTACGATGGCTTTTAGA
sgNick_Q24_OT3	CAAGTGTACTTTCATCAGAACAGG
sgNick_Q24_OT3_G1F	GATACTCACAGGGTTCTGTG
sgNick_Q24_OT3_G1R	TGCTTCATGCCTGGGCAAAA
sgNick_Q24_OT4	CAACTGAACTGTGAGAGAACAGG
sgNick_Q24_OT4_G1F	GTCCTTTATGCCACTGTCGC
sgNick_Q24_OT4_G1R	CAATGAGGCTCGAGCTTTCA
sgNick_Q24_OT5	CAATTGAACTTTGATAAAAATGG
sgNick_Q24_OT5_G1F	GCACTCCCTTGGTCTATTTT
sgNick_Q24_OT5_G1R	GACCTTCTTCAGATTCAATG
sgNick_Q24_OT6	CAAGTGAAATGTGATAGAAGAGG
sgNick_Q24_OT6_G1F	TGAACATGCATCCAGGCTAT
sgNick_Q24_OT6_G1R	CGGTAGGCCAACTAAATGTC
sgNick_Q24_OT7	CAAATGAACTTTGAGTAGCACTGG
sgNick_Q24_OT7_G1F	AGAGAGGTTGGAGAAGGGTG
sgNick_Q24_OT7_G1R	CCCAATTCATTGCAGACCAA
sgNick_Q24_OT8	CAAGTGGAGCTTTGATAGACCAGG
sgNick_Q24_OT8_G1F	GTCACTCATGCACACATTTA
sgNick_Q24_OT8_G1R	CATTTGTTTCAGCCCTTGAA
sgNick_Q24_OT9	TAAGTGAACTTTAATAAGAACAGG
sgNick_Q24_OT9_G1F	TGCATGTGTGTGCGTGCATG
sgNick_Q24_OT9_G1R	AAAAACAAAGGAGGCCGATT
sgNick_Q24_OT10	CAAGTGGAACTTTTATAGAACAGG
sgNick_Q24_OT10_G1F	AGGCCCTTGTTCTTTCACTC
sgNick_Q24_OT10_G1R	GCAGGTGGAACTTTTTTTTT
peg353del-OT1	ACAGCTTGGCAGCTGGGGAAAGG
peg353del-OT1_G1F	ACAGACTTTCAATTTTGCAC

peg353del-OT1_G1R	GCTGCATGAATGAAAGAAAT
peg353del-OT2	GCAGCTTGGGAAGTGGGGAAGGG
peg353del-OT2_G1F	TGCTCTACCCTGATGCTCTG
peg353del-OT2_G1R	ACGAACAGAAGACGCTTCCA
peg353del-OT3	ACAGCTTTGGAACTGGGTAATGG
peg353del-OT3_G1F	TCTTGAATGCTTTGCTGCTT
peg353del-OT3_G1R	TTAAAAGAGCCTGGGACCTC
peg353del-OT4	AGAGCTTGGGACCTGCGGAAGGG
peg353del-OT4_G1F	TGTCATTTGCACTGGAAGGC
peg353del-OT4_G1R	CCTGCCTCGTGTGTGGTTGG
peg353del-OT5	ACAGCTTTGGAACTGGGTAATGG
peg353del-OT5_G1F	TTTCACAGATCCCTAGGGCT
peg353del-OT5_G1R	TTCCCTTGGTGCTGTTCTTG
peg353del-OT6	ACAGTTTGGGACCTGAGAAACGG
peg353del-OT6_G1F	TACGATAAGTGAAGTCAGAG
peg353del-OT6_G1R	GGATGCTAAATTAAGATACG
peg353del-OT7	ACAGTTTGGGTCCTGGGGAGTGG
peg353del-OT7_G1F	TTCTGGGCATGCCAGTCCTT
peg353del-OT7_G1R	TTCTTCTGCAGAGAGGGGTC
peg353del-OT8	ACAGATTGGGGGCTGGGGAATGG
peg353del-OT8_G1F	TGCCTACTATGTCCTAAGAT
peg353del-OT8_G1R	TGATGCTGTCTATTCCACTC
peg353del-OT9	AGAGCTTGGCAGCTGGGGAATGG
peg353del-OT9_G1F	GTGCTAGACCCTGGAGAGAC
peg353del-OT9_G1R	CTAGCAGCTCACCTTCCTCT
peg353del-OT10	AAAGCTTGTGAGCTGGGGAAGGG
peg353del-OT10_G1F	AAAATTCATTCTCCCGGCCT
peg353del-OT10_G1R	GGCAAGACAGTAGGGTAAAG
sgNick353del-OT1	ATTTCCCTGGGTCCCTTAGAAAGG
sgNick353del-OT1_G1F	TTGAGGTGCAGAACAGAAGC
sgNick353del-OT1_G1R	GAGTGGCATAAGGCCTGGGG
sgNick353del-OT2	ATTTCCTGTGCCTGTTAGGCATGG
sgNick353del-OT2_G1F	TACTTCTGGCTCCACTTTCA
sgNick353del-OT2_G1R	AGGAGAGAGGGAGGACTAAT
sgNick353del-OT3	ATCTTACTGGGTCCCCTAGCAGGG
sgNick353del-OT3_G1F	CCTCCTGTGAAATGGGAACA
sgNick353del-OT3_G1R	TCATTATGCCCCTCCTTCCA
sgNick353del-OT4	ATTTCCTGGGTTCCGGTGGTATGG
sgNick353del-OT4_G1F	TCCATAAGGCCCACCAGAAG
sgNick353del-OT4_G1R	AGAGCCTTCTCCTGTTCTGG
sgNick353del-OT5	ATTTCCCTGGGTCCCTTAGAAAGG
sgNick353del-OT5_G1F	ATAAGGCCTGGGGTAGAATA

sgNick353del-OT5_G1R	TTGAGGTGCAGAACAGAAGC
sgNick353del-OT6	TTTTCCTGAGTCCGTTAGGAATGG
sgNick353del-OT6_G1F	TGTTACCTGGACAGAACCAA
sgNick353del-OT6_G1R	GGGCAAAACTCCGCCTCAAA
sgNick353del-OT7	ATTTCCTGTGTTCGTTTGCTAAGG
sgNick353del-OT7_G1F	AGACAGTGTGACAATACCTC
sgNick353del-OT7_G1R	TTTTCTCTGTTGAAACCGAC
sgNick353del-OT8	ATTTCCTGGGGCAGGTGAGCAGGG
sgNick353del-OT8_G1F	GCCTTTCCTGTAAGCAGCTG
sgNick353del-OT8_G1R	GCTGATGATGATCAAGCTGG
sgNick353del-OT9	ATTGTCCTGGGGCCGTCAGCCAGG
sgNick353del-OT9_G1F	ACATAAGTGCCAGAAGCGAA
sgNick353del-OT9_G1R	GTGAAATGTTCAACGTGTCT
sgNick353del-OT10	ATTTCACTGGGTCCTTTGGAAAGG
sgNick353del-OT10_G1F	TACCTTCCTACCAGCCCAGA
sgNick353del-OT10_G1R	AACCATCTCACAGTCAGGAA
SARS-CoV-2-N-F	GGGGAACTTCTCCTGCTAGAAT
SARS-CoV-2-N-R	CAGACATTTTGCTCTCAAGCTG
SARS-CoV-2-N-probe	FAM-TTGCTGCTGCTTGACAGATT-TAMRA

Table S2 Calculation of on-target ACE2 codon substitutions and deletions.

Table S3 Calculation of editing frequency at peg/sgRNA-dependent OT site.

Table S4 ACE2 enzyme activity.

Table S5 Luminescence representing pseudovirus entry.

Table S6 Live virus genomic copies representing virus entry (supernatants).

Table S7 pegRNA sequences.
